# Enhancing the Efficacy of Metal‐Free MRI Contrast Agents via Conjugating Nitroxides onto PEGylated Cross‐Linked Poly(Carboxylate Ester)

**DOI:** 10.1002/advs.202000467

**Published:** 2020-06-03

**Authors:** Shiwei Guo, Xiaoming Wang, Yan Dai, Xinghang Dai, Zhiqian Li, Qiang Luo, Xiuli Zheng, Zhongwei Gu, Hu Zhang, Qiyong Gong, Kui Luo

**Affiliations:** ^1^ Huaxi MR Research Center (HMRRC), Department of Radiology, Functional and molecular imaging Key Laboratory of Sichuan Province, National Clinical Research Center for Geriatrics, State Key Laboratory of Biotherapy, West China Hospital Sichuan University Chengdu 610041 P. R. China; ^2^ Department of Pharmacy of the Affiliated Hospital of Southwest Medical University Southwest Medical University Luzhou Sichuan Province 646000 P. R. China; ^3^ Department of Radiology, Chongqing General Hospital University of Chinese Academy of Sciences (UCAS) 104 Pipashan Zheng Street Chongqing 400014 P. R. China; ^4^ West China School of Medicine Sichuan University Chengdu 610041 P. R. China; ^5^ Amgen Bioprocessing Centre Keck Graduate Institute Claremont CA 91711 USA

**Keywords:** branched polymers, magnetic resonance imaging, metal‐free contrast agents, nitroxides, poly(carboxylate ester)

## Abstract

Herein, two water‐soluble PROXYL‐based magnetic resonance imaging (MRI) macromolecular organic contrast agents (mORCAs) are designed and synthesized: linear and cross‐linked PCE‐mPEG‐Ppa‐PROXYL. They are prepared by conjugating linear and cross‐linked poly(carboxylate ester) (PCE) with poly(ethylene glycol) (mPEG_2000_)‐modified nitroxides (PROXYL), respectively. Both mORCAs form self‐assembled aggregates in an aqueous phase and PROXYL is protected inside a hydrophobic core to achieve great resistance to reduction in the physiological environment, and they have low toxicity. Since cross‐linked PCE‐mPEG‐Ppa‐PROXYL possess a branched architecture, its self‐assembled aggregate is more stable and compact with a greater particle size. Cross‐linked PCE‐mPEG‐Ppa‐PROXYL outperform the linear one in the following aspects: 1) its longitudinal relaxivity (*r*_1_ = 0.79 mm ^−1^ s^−1^) is higher than that of the linear one (*r*_1_ = 0.64 mm ^−1^ s^−1^) and both excel the best mORCA reported so far (*r*_1_ = 0.42 mm ^−1^ s^−1^); 2) its blood retention time (≈48 h) is longer than that of its linear counterpart (≈10 h); 3) cross‐linked PCE‐mPEG‐Ppa‐PROXYL provided better MR imaging contrast resolution in normal organs (liver and kidney) and tumor of mice than the linear one. Overall, cross‐linked PCE‐mPEG‐Ppa‐PROXYL may have great potential to be a novel metal‐free macromolecular contrast agent for MR imaging.

## Graphical abstract

Two biodegradable PEGylated and nitroxides functionalized poly(carboxylate ester) are prepared. The cross‐linked polymer is superior to the linear one in terms of longitudinal relaxivity (0.79 vs 0.64 mm ^−1^ s^−1^) and MR imaging in liver and tumor of mice, so it shows great potential to be a novel metal‐free macromolecular contrast agent for MR imaging.

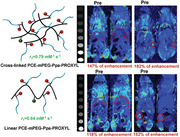

Metal‐based contrast agents (CAs) are commonly used in MR imaging for enhancing imaging sensitivity, nevertheless, they encounter serious toxic issues due to metal ions.^[^ ^1^ ^]^ Therefore, there is a pressing need for developing safe and effective metal‐free MRI CAs. It has been demonstrated that stable nitroxides (e.g., 2,2,5,5‐tetramethyl‐1‐pyrrolidinyl‐N‐oxyl (PROXYL), 2,2,6,6‐tetramethylpiperidinyl‐1‐oxyl (TEMPO), etc.) with unpaired electrons were capable of offering MR imaging contrast via shortening the relaxation of ^1^H protons of water.^[^ ^2^ ^]^ However, commonly‐used nitroxides with a low molecular weight (MW) could not be applied clinically. First, nitroxides have only one unpaired electron, thus they have a relatively low longitudinal relaxivity (the *r*_1_ value), while in metal‐based CAs, gadolinium (Gd^3+^) has seven unpaired electrons and manganese (Mn^2+^) has five ones.^[^ ^3^ ^]^ Second, nitroxides are sensitive to endogenous reducing species (e.g., glutathione and ascorbate), and they can be rapidly reduced to diamagnetic hydroxylamines in vivo.^[^ ^3^, ^4^ ^]^ Therefore, commonly‐used nitroxides fail to provide clinically applicable contrast within a clinically relevant time scale.

According to previous reports, conjugation of small molecular CAs with macromolecules is an effective strategy to improve in vitro and in vivo imaging efficacy of CAs.^[^ ^5^ ^]^ To date, this strategy also has been applied for constructing nitroxides‐based macromolecular organic contrast agents (mORCAs) to overcome the above shortcomings of nitroxides.^[^ ^3^, ^6^ ^]^ Crowds of low‐MW nitroxides are covalently conjugated to macromolecular scaffolds, thus the relatively low relaxivity of nitroxides is increased in multiple orders. The pioneering study was to conjugate low‐MW nitroxides onto terminal functional sites of poly(propyleneimine) or poly(amidoamine) dendrimers.^[^ ^5^, ^6^ ^]^ It has been demonstrated that the *r*_1_ values of these nitroxides‐based mORCAs were proportional to the number of nitroxides per macromolecule. The bioreduction rate of nitroxides, in comparison with free nitroxides, was significantly decreased.^[^ ^5^, ^6^, ^7^ ^]^ However, poor water solubility and biodegradability of these mORCAs hamper their applications. Rajca et al. introduced poly(ethylene glycol) (PEG) chains into spirocyclohexyl nitroxides‐based polypropylenimine dendrimers, resulting in good water solubility, an adequate half‐life of nitroxides and a high *r*_1_ value (0.42 mm ^−1^ s^−1^).^[^ ^8^ ^]^ The same strategy was employed to prepare spirocyclohexyl nitroxides‐based branched‐bottle brush polymeric mORCAs (ORCAFluor P1)^[^ ^9^ ^]^ and brush‐arm star polymeric mORCAs (BASP‐ORCAs).^[^ ^3^, ^10^ ^]^ As expected, similar results were obtained. However, several challenges still remain, including: 1) poor biodegradability of macromolecules, resulting in potential toxicity (e.g., reactive oxygen species (ROS) effects, inflammation, and neuronal toxicity);^[^ ^11^ ^]^ 2) complexity in synthesizing these macromolecules. Two biodegradable nitroxides‐based mORCAs, a PROXYL‐based amphiphilic poly(ethylene glycol)‐b‐polycarbonate (PEG‐b‐PC) diblock copolymer (P1)^[^ ^12^ ^]^ and a TEMPO‐based water‐soluble polyurethane mORCA (PU6‐ORCA),^[^ ^13^ ^]^ have been prepared to address these challenges via simple synthesis methods. They were biodegraded to low‐MW segments in the physiological medium. Their *r*_1_ values may be improved (0.22 mm ^−1^ s^−1^ for P1 and 0.19 mm ^−1^ s^−1^ for PU6‐ORCA). A low loading of nitroxides was the primary reason for such low *r*_1_ values for both biodegradable nitroxides‐based mORCAs. Recently, self‐assembled bi(2,2,6,6‐tetramethyl‐3,6‐dihydropyridin‐1‐oxyl, TEMDO)‐modified ureabenzene nanoparticles (2‐HEG) have been prepared, and this bi(TEMDO)‐based CA had a high loading of nitroxides and a high *r*_1_ value (0.41 mm ^−1^ s^−1^). It was not attempted for in vivo MR imaging.^[^ ^14^ ^]^ Therefore, we hypothesize that more efficient nitroxides‐based mORCAs could be obtained via manipulating key properties of polymers including MW, particle size, load capacity, solubility, and biodegradability via different preparation methods.

Herein, we designed and prepared water‐soluble PROXYL‐based poly(carboxylate ester) MRI mORCAs from the same monomer but with different structures (linear and cross‐linked PCE‐mPEG‐Ppa‐PROXYL, see **Figure** **1**), which consisted of nontoxic components. Because the cross‐linked structure may be more rigid than the linear one, cross‐linked PCE‐mPEG‐Ppa‐PROXYL could form a stable self‐assembled aggregate (Figure 1). Therefore, PROXYL could be more effectively protected inside the hydrophobic core and higher in vivo stability could be achieved.

**Figure 1:**
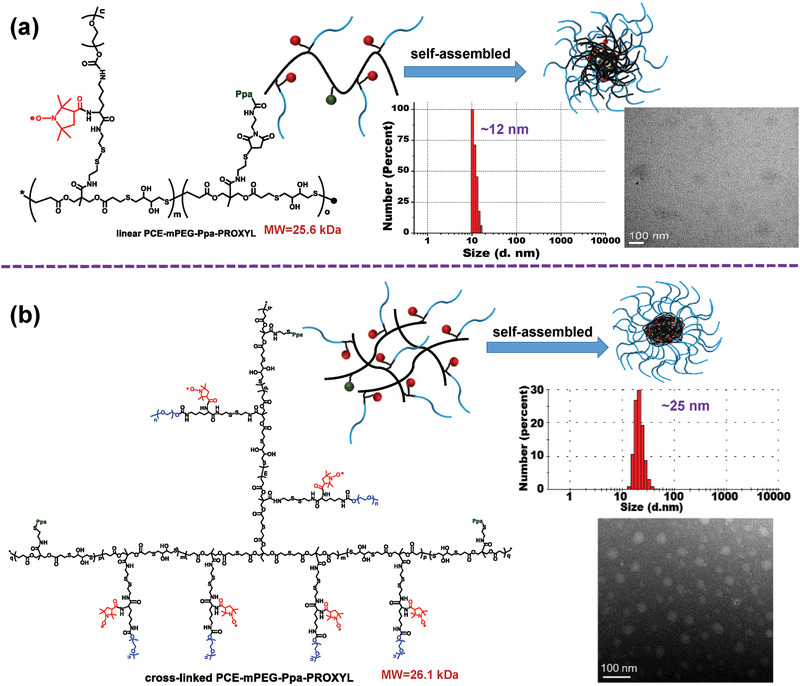
Illustration of linear a) and cross‐linked b) PCE‐mPEG‐Ppa‐PROXYL. DLS results and SEM images for linear and cross‐linked PCE‐mPEG‐Ppa‐PROXYL were embedded in the illustrations.

The synthesis process for both linear and cross‐linked PCE‐mPEG‐Ppa‐PROXYL was quite simple (Schemes S1–S3, Supporting Information). First, we prepared diacrylate (DAC) monomer with thiols protected by 4‐Methoxytrityl (Mmt) and PEGylated PROXYL‐based polymer (PTE‐mPEG‐PROXYL), respectively (Scheme S1, Supporting Information). The cross‐linked polymer with Mmt groups (cross‐linked PCE‐SMmt) was synthesized via amine‐catalyzed thiol‐ene click polymerization of dithithreitol (DTT) with DAC‐SMmt using 10% of 1,1,1‐propanetrimethanol tris(3‐mercaptopropionate) (TMTP) as a cross‐linker. After polymerization, the cross‐linked PCE‐SMmt was treated in an acidic environment (CF_3_COOH/Et_3_SiH) to remove the Mmt groups, and a fluorescence dye (maleimide functionalized pyropheophorbide‐αα, Ppa‐maleimide Ppamaleimide) and PTE‐mPEG‐PROXYL were successively conjugated onto the polymeric branched chains, resulting in cross‐linked PCE‐mPEG‐Ppa‐PROXYL (Scheme S2, Supporting Information). The preparation process for the linear PCE‐mPEG‐Ppa‐PROXYL was similar to that of the cross‐linked one except no cross‐linking agent in the polymerization (Scheme S3, Supporting Information).

The diacrylate monomer with a MmtS group (DAC‐SMmt) was synthesized via a condensation reaction (Scheme S1, Supporting Information), and its structure was confirmed by ^1^H nuclear magnetic resonance spectra (NMR), ^13^C NMR, liquid chromatography‐mass spectrometer (LC‐MS) and matrix assisted laser desorption ionization high resolution mass spectrometry (MALDI‐HR MS) (Figures S1–S4, Supporting Information). In order to simultaneously achieve good water solubility and a high loading of PROXYL, a low‐MW polymer at a ratio of 1:1 between mPEG_2000_ chains and PROXYL (PTE‐mPEG‐PROXYL) were used in the synthesis procedure. The synthesis of PTE‐mPEG‐PROXYL was performed in the following steps, as shown in Scheme S1 (Supporting Information). First, a condensation reaction between 2‐(pyridin‐2‐yldisulfanyl)ethan‐1‐amine hydrochloride (PTE·HCl) and Boc‐Lys(Fmoc)‐OH was carried out, resulting in PTE‐NHBoc with a dithiopyridine group, and this was evidenced from the spectra of ^1^H NMR, ^13^C NMR, LC‐MS and MALDI‐HR MS (Figures S5–S8, Supporting Information). Next, PTE‐NHBoc was treated with CF_3_COOH to remove the Boc group followed by its amidation with 3‐carboxyl‐PROXYL (3‐CP), resulting in an intermediate product PTE‐PROXYL, which was confirmed by LC‐MS and MALDI‐HR MS (Figures S9 and S10, Supporting Information). Additionally, the peaks in the ^1^H NMR spectrum (Figure S11, Supporting Information) became too broad to be quantified, which indicated the presence of paramagnetic nitroxides.^[^ ^8^, ^15^ ^]^ Due to poor stability of the dithiopyridine group in a strong organic base (e.g., piperidine), deprotection (de‐Fmoc) of PTE‐PROXYL was realized under carefully controlled conditions (piperidine, 4 °C, 30 min). The deprotected PTE‐PROXYL reacted with mPEG‐BnNO_2_, yielding PTE‐mPEG‐PROXYL. Compared with the ^1^H NMR spectrum of PTE‐PROXYL, the intensity of peaks in the low field region was clearly reduced, while the intensity of peaks ranging from 2.5 to 5.0 ppm was significantly boosted as shown in Figure S12 (Supporting Information). The MW was larger compared to mPEG‐BnNO_2_ as shown in Figure S13 (Supporting Information). These results indicated that removal of the Fmoc groups and subsequent introduction of mPEG_2000_ chains were successfully accomplished.

It have been demonstrated that amine‐catalyzed thiol‐ene click polymerization is an effective and simple strategy to obtain various functionalized polymers.^[^ ^16^ ^]^ Herein, to achieve good degradability of nitroxides‐based mORCAs, both the diacrylate monomer (DAC‐SMmt) and the tri‐thiol cross‐linker (TMTP) contained hydrolyzable ester bonds in the backbone. Additionally, the amount of the cross‐linker (TMTP) in the mORCAs should be controlled to be no greater than 10%, because if the amount exceeded, the solubility of the polymer became very poor. The amine‐catalyzed thiol‐ene click polymerization of DAC‐SMmt and DTT with 10% TMTP as a cross‐linking agent was performed at 25 °C for only 10 min, resulting in a cross‐linked polymer with Mmt groups (cross‐linked PCE‐SMmt, Scheme S2, Supporting Information). Compared with ^1^H NMR spectrum of DAC‐SMmt (Figure S1, Supporting Information), the peaks in the ^1^H NMR spectrum (Figure S14, Supporting Information) became wide, and the heights of peaks ranging from 5.6 to 6.5 ppm assigned to the double bonds of DAC‐SMmt were clearly decreased, while the peak heights ranging from 2.0 to 5.0 ppm were increased, indicating successful polymerization of DAC‐SMmt and DTT. In addition, gel permeation chromatography (GPC) analysis showed that this polymer had a MW of 20.0 kDa (Figure S15, Supporting Information). Linear PCE‐SMmt was also obtained via the above polymerization procedure while no cross‐linking agent was used (Scheme S3, Supporting Information). The changes in ^1^H NMR spectra before and after linear polymerization were similar to those for cross‐linking polymers (Figure S16, Supporting Information). The MW of linear PCE‐SMmt was 22.1 kDa (Figure S17, Supporting Information). Additionally, the polydispersity index (PDI) of cross‐linked PCE‐SMmt and linear PCE‐SMmt was 1.2 (Figure S15, Supporting Information) and 1.1 (Figure S17, Supporting Information), respectively.

After the above polymerization, the cross‐linked PCE‐SMmt was treated in CF_3_COOH/Et_3_SiH to remove the Mmt groups, resulting in cross‐linked PCE‐SH. A fluorescence dye (maleimide‐functionalized pyropheophorbide‐*α*, Ppa‐maleimide) for in vivo or in vitro fluorescence imaging was then covalently attached to the branched chain of cross‐linked PCE‐SH via thiol‐ene click chemistry. Because the water solubility of the polymer was very poor, hydrophilic modification was required, but at the same time, the loading amount of nitroxides needed to be guaranteed. Therefore, a PEGylated PROXYL derivative (PTE‐mPEG‐PROXYL) with a 1: 1 ratio of PEG to nitroxides was conjugated onto the branched chains of the polymer via thiol‐disulfide exchange reaction, forming the water soluble cross‐linked PCE‐mPEG‐Ppa‐PROXYL as a green solid. Compared with the ^1^H NMR spectra of PTE‐mPEG‐PROXYL (Figure S12, Supporting Information) and cross‐linked PCE‐SMmt (Figure S14, Supporting Information), the peaks in ^1^H NMR spectrum (Figure S18, Supporting Information) changed significantly in both high and low fields. In addition, the polymeric MW increased from 20.0 to 26.1 kDa after chemical modification and remained low at PDI (1.2) (Figure S19, Supporting Information). As expected, electron paramagnetic resonance (EPR) analysis (Figure S20, Supporting Information) showed that this final polymer possessed paramagnetism and the spin concentration was 0.135 mmol g^−1^. These results indicated that successfully formation of cross‐linked PCE‐mPEG‐Ppa‐PROXYL. Linear PCE‐mPEG‐Ppa‐PROXYL with a MW of 25.6 kDa (Figure S21, Supporting Information) and a spin concentration of 0.072 mmol g^−1^ (Figure S22, Supporting Information) was successfully obtained via the similar procedure. It was soluble in water rather than DMSO due to a high content of hydrophilic components, and its chemical structure was confirmed by ^1^H NMR in D_2_O (Figure S23, Supporting Information). According to the above data, the spin concentration of cross‐linked PCE‐mPEG‐Ppa‐PROXYL were higher than that of the linear one, which implied that cross‐linked PCE‐mPEG‐Ppa‐PROXYL had a higher *r*_1_ values than the linear one.

The particle size of linear and cross‐linked PCE‐mPEG‐Ppa‐PROXYL was directly measured via dynamic light scattering (DLS) and their morphology via transmission electron microscopy (TEM). DLS results showed that two mORCAs could self‐assemble in the aqueous phase to form aggregates via the hydrophilic and hydrophobic effect (Figure 1). However, the particle size and stability of both self‐assembled aggregates were significantly different. The particle size of cross‐linked PCE‐mPEG‐Ppa‐PROXYL (≈25 nm) was significantly higher than that of the linear one (≈12 nm). The morphology of the cross‐linked mORCA was observed under a TEM, while the linear mORCA could not be detected (Figure 1). The structure of cross‐linked PCE‐mPEG‐Ppa‐PROXYL was rigid and its self‐assembled aggregate was very stable, so its morphology could be maintained even after water was removed. However, linear PCE‐mPEG‐Ppa‐PROXYL with a flexible structure formed a loose self‐assembled aggregate, and the structure became scattered after losing the hydrophobic effect due to water removal, thus its morphology could not be observed under a TEM.

In addition, the zeta potential of the linear and cross‐linked mORCAs was close to a neutral value (Figure S24, Supporting Information), which indicated that adsorption of opsonization proteins in the blood onto mORCAs may be avoided, thereby reducing the clearance of both mORCAs by the reticuloendothelial system, ensuring their stability in the blood circulation, and prolonging their circulation time. Moreover, their particle size in the aqueous phase was greater than 10 nm, and they could be effectively accumulated into tumor tissues via the enhanced permeate and retention (EPR) effect, especially for cross‐linked PCE‐mPEG‐Ppa‐PROXYL. Additionally, we used GPC to study the in vitro esterase‐catalyzed degradability (hydrolysis) of the two mORCAs (Figures S25 and S26, Supporting Information). The two polymers showed biodegradable features.

We evaluated the feasibility of cross‐linked PCE‐mPEG‐Ppa‐PROXYL and linear PCE‐mPEG‐Ppa‐PROXYL as MRI contrast agents by measuring their longitudinal relaxivity (*r*_1_) using a clinical 3.0 T MRI scanner. Both cross‐linked PCE‐mPEG‐Ppa‐PROXYL and linear PCE‐mPEG‐Ppa‐PROXYL revealed bright signals in the *T*_1_‐weighted MR imaging. As shown in **Figure** **2**a, the *r*_1_ value was calculated by plotting the 1/*T*_1_ value with gradient concentrations of linear and cross‐linked PCE‐mPEG‐Ppa‐PROXYL. The *r*_1_ value of the cross‐linked PCE‐mPEG‐Ppa‐PROXYL (0.79 mm ^−1^ s^−1^) was significantly increased compared to linear PCE‐mPEG‐Ppa‐PROXYL (0.64 mm ^−1^ s^−1^) and 3‐CP (0.19 mm ^−1^ s^−1^) (Figure 2b). Compared with 3‐CP, two polymers had a greater MW and contained many nitroxides, so the longitudinal relaxivity was significantly increased by the multiplication effect.^[^ ^17^ ^]^ Between two polymers, the nitroxides content (Spin concentration) of the cross‐linked PCE‐mPEG‐Ppa‐PROXYL was higher than that of the linear one, resulting in increasing the local concentration of nitroxides, thus maximizing the multiplication effect. In addition, compared with the linear one, the self‐assembled aggregate from cross‐linked PCE‐mPEG‐Ppa‐PROXYL had a larger nano size, which could delay its flipping speed more effectively to achieve a higher longitudinal relaxivity. Although the self‐assembled aggregates from the cross‐linked polymer were more rigid than those from the linear one, they still had enough flexibility to meet full and rapid contact between the single electron of PROXYL and the surrounding water, therefore, the structural rigidity had no adverse effect on the relaxivity of the cross‐linked PCE‐mPEG‐Ppa‐PROXYL.

**Figure 2:**
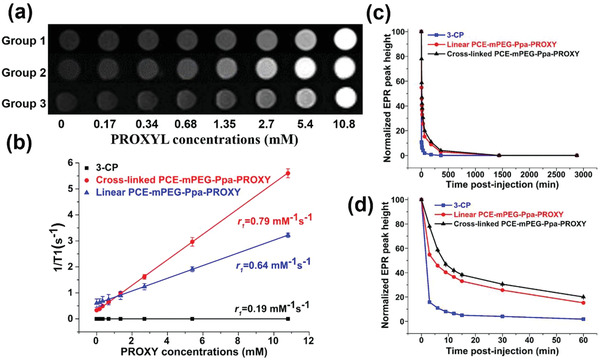
In vitro longitudinal relaxivity a) of linear (Group 1) and cross‐linked PCE‐mPEG‐Ppa‐PROXYL (Group 2) and 3‐CP (Group 3). b) *r*_1_ values of contrast agents. c) In vivo temporal nitroxide concentration profile of linear and cross‐linked PCE‐mPEG‐Ppa‐PROXYL and 3‐CP in the blood up to 48 h, and d) detailed concentration change within 1 h.

We compared metabolism of nitroxides in linear and cross‐linked PCE‐mPEG‐Ppa‐PROXYL and 3‐CP in vivo through electron paramagnetic resonance (EPR) analysis of mouse blood at different time points. The results showed that the concentration of nitroxides of 3‐CP in the blood decreased faster than that of two mORCAs, and was undetectable after injection 1 h (Figure 2c,d), which indicated that 3‐CP was rapidly reduced or metabolized in the body. For two mORCAs, the concentration of nitroxides decreased rapidly in the first 10 min after injection (Figure 2d), and then the speed of decline slowed down significantly (Figure 2c,d). In the linear PCE‐mPEG‐Ppa‐PROXYL group, the blood concentration of nitroxides was still detectable until 10 h after injection (Figure 2c,d), while in the cross‐linked PCE‐mPEG‐Ppa‐PROXYL group, a longer blood circulation time up to 48 h was observed (Figure 2c,d). In the early stage after injection, the concentration of nitroxides was high, so the concentration of nitroxides in the blood decreased at a faster rate. After then slow metabolism of macromolecules and their protective effect on nitroxides gradually played a leading role in maintaining the stability of nitroxides, so the blood concentration of nitroxides in macromolecules decreased slowly. The cross‐linked PCE‐mPEG‐Ppa‐PROXYL self‐assembled in an aqueous environment to form a big (≈25 nm) and compact self‐assembled aggregate, and the hydrophobic nitroxides was better protected inside the hydrophobic core to improve the stability of nitroxides because the contact between endogenous reducing substances and nitroxides in macromolecules could be significantly reduced. However, the self‐assembled aggregate of linear PCE‐mPEG‐Ppa‐PROXYL was smaller (≈12 nm) and loose, and the aggregate could not effectively protect hydrophobic nitroxides, so that nitroxides was rapidly reduced by the endogenous reducing substances in vivo. Additionally, since the metabolic rate of substances in human body was slower than that in mice, it may be expected that the cross‐linked PCE‐mPEG‐Ppa‐PROXYL will have longer retention of nitroxides in human blood, which may result better MR imaging effect in vivo.

We further investigated the in vivo efficacy of both linear and cross‐linked PCE‐mPEG‐Ppa‐PROXYL as a magnetic resonance contrast agent in tumor and healthy major organs of mice. The enhancement in MR signal intensities was quantified at various time points after injection of both mORCAs, and 3‐CP was used as a control.

For MR contrast imaging of major organs, ten healthy BALB/c mice were inspected under a clinical Siemens 3.0 T MRI scanner at different time points from pre‐injection to 0.5 h post‐injection. The MRI signals of major organs (liver and kidney) were enhanced after 5 min of injection, and the trends in the enhancement were different for two mORCAs. In the liver after injection of the cross‐linked PCE‐mPEG‐Ppa‐PROXYL (**Figure** **3**a), the MRI signal enhancement began at 5 min, and reached a peak at 15 min. The enhancement was maintained up to 25 min, and started to decrease down to the pre‐injection level at 30 min. In the kidney (Figure S27, Supporting Information), the MRI signal intensity was incrementally increased up to 30 min post injection of the contrast agent. At 30 min, the signal was much stronger in the kidneys in the cross‐linked PCE‐mPEG‐Ppa‐PROXYL‐treated group. Quantitative analysis of MRI images in the liver and kidney showed that the trend of the *T*_1_ values was different in two organs (Figure 3c and Figure S28, Supporting Information). In the liver, the *T*_1_ value increased by about 147% at 15 min (Figure 3e), after that, it gradually decreased and returned to the pre‐injection level at 30 min. However, in the kidney, the *T*_1_ value substantially increased, compared with the pre‐enhancement level, and reached about 160% at 30 min (Figure S29, Supporting Information).

**Figure 3:**
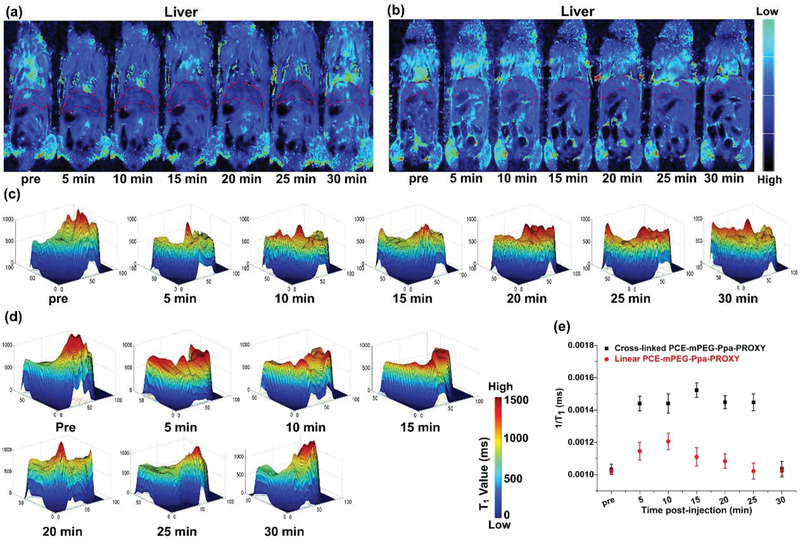
*T*_1_ mapping imaging of liver after injection of a) cross‐linked and b) linear PCE‐mPEG‐Ppa‐PROXYL. The liver was labeled with red dashed lines, and darker blue signals in the liver suggest sharper enhancement in the MR images. In the cross‐linked PCE‐mPEG‐Ppa‐PROXY‐treated group, pronounced enhancement in the liver was achieved after 25 min, while in the linear PCE‐mPEG‐Ppa‐PROXYL‐treated group, signal enhancement in the liver was seen after 15 min but the blue signals are relatively weak. The corresponding *T*_1_ values were spatially displayed in the liver after injection of c) cross‐linked and d) linear PCE‐mPEG‐Ppa‐PROXYL at different durations. e) The 1/*T*_1_ values for two liver groups were quantitatively analyzed (*p *< 0.05).

After injection of the linear PCE‐mPEG‐Ppa‐PROXYL (Figure 3b,d), the MRI signal in the liver started to increase at 5 min, and reached a peak (about 118% of the pre‐injection level) at 10 min (Figure 3e), after that, the signal experienced a decrease, and returned to the pre‐injection MRI signal at 30 min. In the kidney (Figures S30 and S31, Supporting Information), the MRI signal intensity was observed to become stronger within 10 min, and became the strongest (about 120% of the pre‐injection level) at 10 min (Figure S29, Supporting Information). The enhancement was weakened after 10 min. Both organs after injection of 3‐CP were also scanned by the same method as a control, and it was found that there was no obvious enhancement in normal mouse organs (liver and kidney) and no change in the *T*_1_ value (Figure S32, Supporting Information). These results indicated that compared to 3‐CP, two mORCAs had displayed better imaging quality in vivo over a longer time. This could be explained by that two mORCAs had a higher relaxivity as shown from in vitro measurements. Another contributing factor was the stability of PROXYL that was protected in the hydrophobic core of the self‐assembled aggregates of two mORCAs by reducing exposure of nitroxides to endogenous reducing substances. Additionally, between two mORCAs, the cross‐linked PCE‐mPEG‐Ppa‐PROXYL outperformed the linear one in providing stable MRI enhancement in the liver and kidney because of a higher relaxivity of cross‐linked PCE‐mPEG‐Ppa‐PROXYL in vitro and a larger particle size and a more rigid structure of its self‐assembled aggregate. The in vivo imaging results of the above three groups were consistent with their in vitro relaxivities. Meanwhile, a continuous increase in the MRI signal and the *T*_1_ value in the kidney indicated that both linear and cross‐linked PCE‐mPEG‐Ppa‐PROXYL were mainly through the metabolism in the kidney, which ensured the safety of the contrast agents.

For contrast imaging in the tumor by linear and cross‐linked PCE‐mPEG‐Ppa‐PROXYL, two mORCAs exhibited a similar trend in the enhancement of contrast imaging: two mORCAs in breast cancer rapidly enhanced the contrast image within 5 min and the enhancement peaked at 5 min, and then the degree of enhancement decreased continuously to a similar level of brightness as pre‐injection at 30 min. It was noted that linear PCE‐mPEG‐Ppa‐PROXYL (**Figure** **4**b) was weaker than cross‐linked PCE‐mPEG‐Ppa‐PROXYL at the peak time (Figure 4a). Quantitative analysis of the above tumor MRI images by the *T*_1_ value showed that the cross‐linked PCE‐mPEG‐Ppa‐PROXYL peaks at the tumor site at around 5 min (Figure 4c), and the *T*_1_ value increased by about 182% (Figure 4e), indicating this mORCA may be applied to tumor diagnosis and imaging. Similarly, the signal enhancement by linear PCE‐mPEG‐Ppa‐PROXYL reached a peak at the tumor site at around 5 min (Figure 4d), but the signal was weaker than cross‐linked PCE‐mPEG‐Ppa‐PROXYL, and the *T*_1_ value increases about 152% (Figure 4e). This result was well correlated with the in vitro relaxivity results of linear and cross‐linked PCE‐mPEG‐Ppa‐PROXYL and aligned well with the MR imaging results in the healthy organs. 3‐CP was used as a control in tumor imaging and it was found that there was no signal enhancement at the tumor site (Figure S33, Supporting Information). Both mORCAs had a nano size of >10 nm and more nitroxides in both polymers were accumulated in the tumor site via the EPR effect.^[^ ^18^ ^]^ Since cross‐linked PCE‐mPEG‐Ppa‐PROXYL had a larger nano size and the EPR effect at the tumor site was much better than the linear one, the degree of enhancement was higher. The MRI signal and the *T*_1_ value of two mORCAs decayed rapidly in the tumor microenvironment in comparison with those in healthy organs, which was due to a high concentration of reducing substances (such as glutathione) in tumor tissues, leading to rapid conversion of nitroxides into diamagnetic nitrogen‐hydroxy compounds under the action of reducing substances.

**Figure 4:**
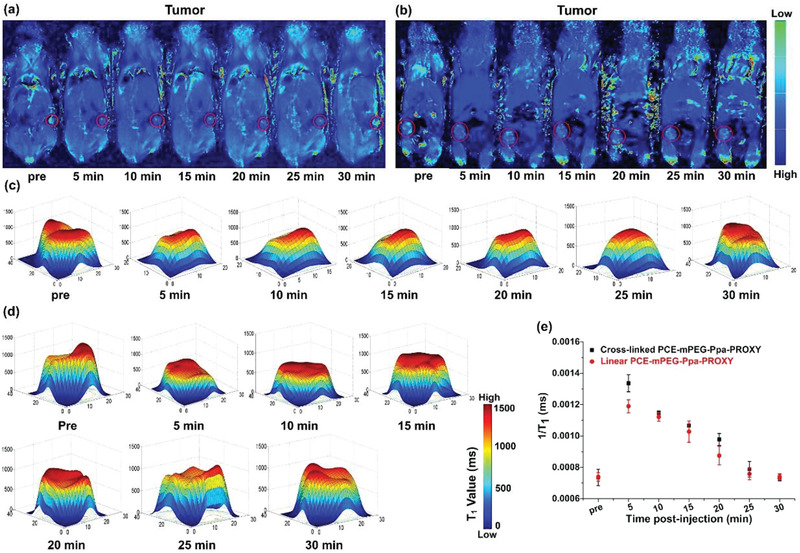
*T*_1_ mapping imaging of tumor by a) cross‐linked and b) linear PCE‐mPEG‐Ppa‐PROXYL. The tumor was labeled with red lines, and darker blue signals in the tumor suggest sharper enhancement in the MR images. Two mORCAs exhibited a similar enhancement trend as the post‐injection time extends, but the degree of enhancement from cross‐linked PCE‐mPEG‐Ppa‐PROXYL was better than the linear one. The corresponding *T*_1_ values were spatially displayed in the tumor after injection of c) cross‐linked and d) linear PCE‐mPEG‐Ppa‐PROXYL at different durations. e) The 1/*T*_1_ values of two tumor groups were quantitatively analyzed (*p *< 0.05).

In addition, the *T*_1_ relaxivity was measured via the MR *T*_1_ mapping sequence method in accordance with the signal intensity by different flip angles. The biggest advantage of this method was that it could sensitively detect changes in the MRI signal caused by the low relaxivity nitroxide magnetic resonance contrast agent and accurately measured changes in the *T*_1_ value from each voxel in the image, and more precisely quantified the degree of enhancement in the signal intensity at the organ or tumor site. To the best of our knowledge, this sequence was used for the first time to evaluate the in vivo imaging of nitroxides‐based magnetic resonance contrast agents. At the same time, using this sequence, we found that cross‐linked PCE‐mPEG‐Ppa‐PROXYL had a better in vivo imaging effect, compared to linear PCE‐mPEG‐Ppa‐PROXYL and 3‐CP.

The linear and cross‐linked PCE‐mPEG‐Ppa‐PROXYL polymers were uptaken by cells but showed low toxicity (Figures S34–S38, Supporting Information). The detailed results and discussion were provided in the Supporting Information.

Herein, PROXYL (a common nitroxide) was conjugated with PEGylated linear and cross‐linked poly(carboxylate ester) to obtain two novel amphiphilic nitroxides‐based mORCAs: linear and cross‐linked PCE‐mPEG‐Ppa‐PROXYL. They formed nano‐sized self‐assembled aggregates in an aqueous environment and PROXYL was protected in the hydrophobic core. Cross‐linked PCE‐mPEG‐Ppa‐PROXYL had a higher spin concentration, a larger particle size and higher stability for nitroxides. Therefore, compared with linear PCE‐mPEG‐Ppa‐PROXYL and reported similar mORCAs, the cross‐linked PCE‐mPEG‐Ppa‐PROXYL possessed a higher longitudinal relaxivity, and provided long‐term and significant in vivo MRI *T*_1_ mapping signal enhancement in tumors or other organs. In addition, cross‐linked PCE‐mPEG‐Ppa‐PROXYL displayed non‐toxicity. Therefore, cross‐linked PCE‐mPEG‐Ppa‐PROXYL, as a new structure of nitroxides‐based macromolecular magnetic resonance contrast agent, may be expected as candidate for metal‐free‐based magnetic resonance contrast agent.

## Conflict of Interest

The authors declare no conflict of interest.

## Supporting information

Supporting Information
