# Supplementary material for: Enhancing the Efficacy of Metal‐Free MRI Contrast Agents via Conjugating Nitroxides onto PEGylated Cross‐Linked Poly(Carboxylate Ester)
Source: Adv Sci (Weinh). 2020 Jun 3;7(14):2000467. doi: 10.1002/advs.202000467 (PMC7375229; doi:10.1002/advs.202000467)
Supplement: Supplementary file 1 — Supporting Information [file ADVS-7-2000467-s001.pdf]

Copyright WILEY-VCH Verlag GmbH & Co. KGaA, 69469 Weinheim, Germany, 2019.

Supporting Information

Enhancing the efficacy of nitroxides-based magnetic resonance imaging contrast agent via conjugating onto a PEGylated cross-linked poly(carboxylate ester)

*Shiwei Guo, Xiaoming Wang, Yan Dai, Xinghang Dai, Zhiqian Li, Qiang Luo, Xiuli Zheng, Zhongwei Gu, Hu Zhang, Qiyong Gong, Kui Luo**

Dr. S. W. Guo, Mr. X. M. Wang, Dr. Y. Dai, Mr. Z. Q. Li, Mr. Q. Luo, Mrs X. L. Zheng, Prof. Z. W. Gu, Prof. Q. Y. Gong, Prof. K. Luo
Huaxi MR Research Center (HMRRC), Department of Radiology, Functional and molecular imaging Key Laboratory of Sichuan Province, National Clinical Research Center for Geriatrics, State Key Laboratory of Biotherapy, West China Hospital, Sichuan University, Chengdu 610041, China
*E-mail: luokui@scu.edu.cn

Mr. X. H. Dai
West China School of Medicine, Sichuan University, Chengdu 610041, China

Dr. S. W. Guo, Dr. Y. Dai
Department of Pharmacy of the Affiliated Hospital of Southwest Medical University, Southwest Medical University, Luzhou, Sichuan Province, 646000, China.

Mr. X. M. Wang
Department of Radiology, Chongqing General Hospital, University of Chinese Academy of Sciences (UCAS), 104 Pipashan Zheng Street, Chongqing 400014, PR China.

Prof. H. Zhang
Amgen Bioprocessing Centre, Keck Graduate Institute, CA 91711, USA

EXPERIMENTAL SECTION

Materials and Measurements: All commercial reagents were used without further purification unless otherwise stated. **TMTP**, **3-CP**, **DTT** and DIC were purchased from TCI (Japan), while **Boc-Lys(Fmoc)-OH**, HBTU, HOBt from GL Biochem (China). **Ppa-maleicimide**, **DAC-COOH**, **MmtS-NH$_2$**, **PTE•HCl,** and **mPEG-BnNO$_2$** were synthesized according to literature.[1-5] The weight-average molecular weight (MW) and polydispersity index (PDI) of the polymers were measured *via* gel permeation chromatography (GPC) on an AKTA/FPLC system (GE Healthcare). All ^{1}H NMR and ^{13}C NMR spectra were recorded on a Bruker 400 (400 MHz) spectrometer. MS spectra were obtained from an electrospray ionization time-of-flight mass spectrometry (ESI-TOF MS, Waters Q-TOF Premier) and a matrix assisted laser desorption ionization time-of-light mass spectrometry (MALDI-TOF, Autoflex MALDI-TOF/TOF). Electron Paramagnetic Resonance (EPR) spectra were acquired using a Bruker EMX plus X-band CW spectrometer. The size and zeta potential were detected by a Zetasizer (Malvern, Worcestershire, U.K.).

Synthesis of DAC-SMmt: Under N_2, DMAP (294 mg, 2.39 mmol) was added to a solution of **DAC-COOH** (5.8 g, 23.87 mmol), DIC (4.5 g, 35.92 mmol) and **MmtS-NH$_2$** (10.0 g, 28.64 mmol) in DCM (100 mL) at ambient temperature. The reaction mixture was stirred at the ambient temperature for overnight, TLC determined the end of reaction. The mixture was concentrated in vacuum to a crude yellow oil, which was further purified by silica gel (petroleum ether/ethyl acetate, 2:1, then petroleum ether/acetone, 3:1) to give **DAC-SMmt** as a light yellow oil (6.7 g, yield: 49.0%). ^{1}H NMR (400 MHz, d_6-DMSO): δ 7.95 (s, 1 H), 7.17-7.33 (m, 12 H), 6.88 (d, J=8.0 Hz, 2 H), 6.24-6.28 (m, 2 H), 6.03-6.10 (m, 2 H), 5.86-5.89 (m, 2 H), 4.15-4.22 (m, 4 H), 3.74 (s, 3 H), 3.05 (s, 2 H), 2.18 (s, 2 H), 1.14 (s, 3 H). ^{13}C NMR

(400 MHz, d_6-DMSO): δ 171.54, 165.08, 157.70, 144.82, 136.27, 131.76, 130.31, 128.96, 127.97, 127.94, 126.64, 113.25, 65.85, 65.36, 55.05, 54.92, 45.46, 37.85, 31.14, 17.52. MS (ESI, *m/z*): 596.2 $[M+Na]^+$.

Synthesis of PTE-NHBoc: Under N_2, HBTU (2.1 g, 5.63 mmol), HOBt (1.0 g, 7.50 mmol) and N,N-Diisopropylethylamine (2.4 g, 18.75 mmol) were added to a solution of **Boc-Lys(Fmoc)-OH** (1.8 g, 3.75 mmol) in DMF (10 mL) at 0 ^{o}C and the mixture was stirred at ambient temperature for 1 h. At 0 ^{o}C, after a solution of **PTE·HCl** (1.0 g, 4.50 mmol) in DMF (3 mL) was added drop-wise into the above reaction mixture over 30 min, the solution was stirred at ambient temperature for overnight, quenched with sat.aq.$NaHCO_3$ (50 mL), extracted with ethyl acetate (50 mL*2). The combined organic layers were washed with sat.aq.$NaHCO_3$ (50 mL*2), 1N HCl (50 mL*2) and brine (50 mL), dried over Na_2SO_4, filtered and concentrated. The crude compound was purified by silica gel (petroleum ether/ethyl acetate, 1:1 to 1:2) to give **PTE-NHBoc** as a white solid (1.7 g, yield: 71.2%). ^{1}H NMR (400 MHz, $CDCl_3$): δ 8.52-8.54 (m, 1 H), 7.73-7.75 (m, 3 H), 7.56-7.60 (m, 3 H), 7.46-7.48 (m, 1 H), 7.35-7.39 (t, *J*=8.0 Hz, 2 H), 7.26-7.30 (m, 2 H), 7.09-7.12 (m, 1 H), 5.20 (brs, 1 H), 4.91 (s, 1 H), 4.36-4.38 (m, 2 H), 4.11-4.20 (m, 2 H), 3.61 (br s, 1 H), 3.46 (br s, 1 H), 3.04-3.20 (m, 2 H), 2.80-2.95 (m, 2 H), 2.02 (brs, 1 H), 1.80-1.90 (m, 1 H), 1.58-1.70 (m, 1 H), 1.45-1.55 (m, 2 H), 1.34-1.44 (m, 10 H). ^{13}C NMR (400 MHz, $CDCl_3$) δ 172.58, 156.89, 156.22, 144.27, 141.65, 137.95, 127.97, 127.31, 125.33, 121.65, 120.57, 80.53, 66.97, 54.81, 47.61, 40.68, 38.94, 37.62, 32.64, 29.79, 28.72, 22.83. MS (ESI, *m/z*): 637.2 $[M+H]^+$, 659.2 $[M+Na]^+$, 675.2 $[M+K]^+$.

Synthesis of PTE-PROXYL: CF_3COOH (5 mL) was added drop-wise to a solution of **PTE-NHBoc** (1.5 g, 2.36 mmol) in DCM (5 mL) at ambient temperature over 10 min, and the reaction mixture was stirred at ambient temperature for 3 h. After

evaporation of solvents, the residual oil was washed with ether (40 mL*2) to give a crude white solid. Under N_2, a solution of **3-CP** (360 mg, 1.96 mmol), DCC (487 mg, 2.36 mmol) and N,N-Diisopropylethylamine (305 mg, 2.36 mmol) in DCM (10 mL) was stirred at ambient temperature for 30 min, and then the above crude white solid was added into the above solution. The reaction mixture was stirred at ambient temperature for overnight, filtered and concentrated to a crude oil. The oil was purified by silica gel (petroleum ether/ethyl acetate, 1:1 to 1:10) to give **PTE-PROXYL** as a yellow solid (1.2 g, yield: 83.7%). MS (ESI, *m/z*): 705.3 $[M+H]^+$, 727.3 $[M+Na]^+$, 743.3 $[M+K]^+$.

Synthesis of PTE-mPEG-PROXYL: At 4°C, piperdine (1 mL) was added drop-wise to a solution of **PTE-PROXYL** (400 mg, 0.56 mmol) in DMF (4 mL), and the mixture was stirred at 4°C for 0.5 h. After reaction, the mixture was washed with petroleum ether (50 mL*3) and ether (50 mL*3) at 4°C to give a crude light yellow solid. Under N_2, N,N-Diisopropylethylamine (320 mg, 2.49 mmol, 3.0 eq) was added to a solution of the above solid and **mPEG-BnNO$_2$** (1.2 g, 0. 56 mmol) in DCM (20 mL) at ambient temperature, and the reaction mixture was stirred at ambient temperature for 2 days. After dialyzing against RO-water (MWCO: 1.0 KDa) for 24 h and freeze-dry, 1.3 g of crude **PTE-mPEG-PROXYL** as a white solid was obtained.

Synthesis of linear PCE-SMmt: At 25 ^{o}C, **DAC-SMmt** (2.0 g, 3.5 mmol), **DTT** (538 mg, 3.5 mmol) and n-hexamine (353 mg, 0.35 mmol) at a catalytic amount were mixed in THF (4.6 mL) and the mixture was stirred for 10 min. The reaction was quenched *via* dropping the solution to ether (60 mL), and white solids appeared. After filtration, the solids were washed with ether (30 mL*3) followed by vacuum drying, resulting in **linear PCE-SMmt** as a white solid (1.8 g, MW=9.3 kDa, PDI=1.80).

Synthesis of linear PCE-mPEG-Ppa-PROXYL: The **linear PCE-SMmt** (930 mg,

0.1 mmol) was dissolved in DCM (45 mL) and at 0 ^{o}C and under nitrogen, Et_3SiH (1.5 mL) and CF_3COOH (2.5 mL) was added to the linear PCE-SMmt solution successively. The reaction solution was continuously stirred for overnight at room temperature, and then concentrated to remove solvents. The concentrated liquid was added drop-wise to ether (170 mL) and a cloudy solution was formed. After filtration, the solids were washed with ether (20 mL*3) followed by vacuum drying to obtain crude **linear PCE-SH** as a white solid which was directly used for the next step without further purification. The crude **linear PCE-SH** (0.1 mmol) was dissolved in DMF (15 mL), and the fluorescent probe modified by maleicimide (**Ppa-maleicimide**) (100 mg) was then added to the above solution at room temperature. After stirring for 6 h, a green clear solution was obtained. After the introduction of **Ppa**, the nitroxide derivative (**PTE-mPEG-PROXYL**) (6.5 g, 2.6 mmol) and AcOH (0.1 mL) at a catalytic amount were added successively at room temperature. The mixture was stirred at room temperature for 2 days to conjugate PROXYL onto the macromolecular carrier. After the above reaction, the reaction solution was quenched by adding it drop-wise to RO-H_2O (100 mL) in an ice-bath, and then the aqueous solution was dialyzed against RO-H_2O (MWCO: 5.0 kDa) for 24 h. After freeze-drying of the dialyzed product, a crude green solid was generated. The crude solid was washed with ether (15 mL*3) to give **linear PCE-mPEG-Ppa-PROXYL** as a green solid (1.4 g, MW=12.4 kDa, PDI=3.00, Spin conc.=0.072 mmol/g).

Synthesis of cross-linked PCE-SMmt: A solution of **DAC-SMmt** (1.8 g, 3.18 mmol), **DTT** (426 mg, 2.76 mmol), **TMTP** (110 mg, 0.276 mmol) and n-hexamine (56 mg, 0.554 mmol) in THF (4.3 mL) was stirred at 25 ^{o}C for 10 min. The reaction solution was added drop-wise to ether (50 mL) to give a white solid. After filtration, the solid was washed with ether (50 mL*2) and dried in vacuum to obtain

cross-linked PCE-SMmt as a white solid (1.5 g, MW=33.6 kDa, PDI=2.64).

Synthesis of cross-linked PCE-mPEG-Ppa-PROXYL: Under N_2, Et_3SiH (1.5 mL) and CF_3COOH (2.5 mL) was added to a solution of **cross-linked PCE-SMmt** (904 mg, 0.025 mmol) in DCM (47.5 mL) at 0 ^{o}C. The mixture was stirred at ambient temperature for overnight. After concentration, the residue was added drop-wise to ether (150 mL), the solution was filtered and the solids were dried in vacuum to give a white solid. Under N_2, to a solution of the above solid (0.03 mmol) in DMF (20 mL) was added **Ppa-maleicimide** (100 mg) at ambient temperature, and the mixture was continuously stirred at the same temperature for 6 h. **PTE-mPEG-PROXYL** (3.2 g, 1.29 mmol) and AcOH (0.1 mL) were added to the above mixture, stirring at ambient temperature for 2 days. After the reaction solution was dialyzed against RO-water (MWCO: 8.0 kDa) for 24 h, the concentrated product was freeze-dried. The crude solid was washed with ether (50 mL*2) and dried in vacuum to give **cross-linked PCE-mPEG-Ppa-PROXYL** as a green solid (1.1 g, MW = 54.2 kDa, PDI=10.8, Spin conc.=0.135 mmol/g).

***In vitro* degradation:** We studied the degradability (hydrolysis) of **linear and cross-linked PCE-mPEG-Ppa-PROXYL** under the catalysis of esterase by referring to the methods previously reported.[6] Linear and cross-linked PCE-mPEG-Ppa-PROXYL were dissolved in 2 mL of saline (pH=7.4), respectively, then 200 U of the esterase from porcine liver was added to each solution. The solutions were incubated at 37 ^{o}C, and 150 uL of samples were taken out at predetermined time points (0 h, 2 h, 6 h, 12 h, 24 h), the samples were freeze-dried and then 500 uL of DMF was added, after filtration, the samples were analyzed by GPC (Waters styragel HT 3 column, DMF as eluent and polystyrene (PS) as standard sample) to obtain the MW changes during the degradation process.

Phantom relaxivity by MRI: The ^{1}H water relaxivity of **linear and cross-linked PCE-mPEG-Ppa-PROXYL** in PBS was measured by a Siemens 3.0 T MRI scanner. **Linear and cross-linked PCE-mPEG-Ppa-PROXYL** at different PROXYL concentrations (0, 0.17, 0.34, 0.68, 1.35, 2.7, 5.4, 10.8 mM) was dissolved in 0.1 M of PBS, the magnetic resonance signal intensity was scanned by the T1 SE sequence using different TR values (25-1000 ms). The measured magnetic resonance signal values were processed using previously reported special calculation formula and analysis methods to obtain the 1/T1 values. The relaxivity value r_1 was calculated by plotting the 1/T1 value as a function of different nitroxides concentrations. In addition, 3-CP at the same concentration was dissolved in 0.1 M of PBS as a control.

In vivo tumor MR imaging: To evaluate the amount of **linear and cross-linked PCE-mPEG-Ppa-PROXYL** and 3-CP accumulated in the tumor, we used an in situ breast cancer model for this study. The model was built using 4T1 cells. Fifteen female 4-6 week-old BALB/c mice (20 ± 2 g) were arbitrarily divided into three groups (n = 5). 4T1 cells (5×10^7 cells) were injected in the right or lower left breast tissue. In one week after injection, the volume of solid breast tumors reached around 10 mm^3. The 3.0 T MRI scanner was used to obtain the contrast-enhanced T_1 mapping sequence. The T_1 mapping sequence parameters were chosen: TR = 15 ms, TE = 2.04 ms, flip angle = 5° /26° , FOV = 156 × 156 mm, slice thickness = 1 mm. Animals were injected **linear and cross-linked PCE-mPEG-Ppa-PROXYL** and 3-CP at a dose of 0.135 mmol PROXYL /kg via the tail vein. At pre-injection, and 5, 10, 15, 20, 25 and 30 min after injection, the MRI T_1 mapping sequences were acquired and the T_1 values at tumor sites were obtained using METALAB software.[7]

In vivo Major Organ MR imaging: Fifteen healthy female 4-6 week-old BALB/c

mice (20 ± 2 g) were arbitrarily divided into three groups (n = 5), and injected with **linear and cross-linked PCE-mPEG-Ppa-PROXYL** and 3-CP at a dose of 0.135 mmol PROXYL /kg through tail vein for in vivo major organ MR imaging respectively. At pre-injection, 5 min, 10 min, 15 min, 20 min, 25 min and 30 min post-injection, the T_1 mapping MR images were obtained using the 3.0 T clinical MR system. The scanning parameters were described as previously reported. The signal intensity in major organs was quantitatively measured by use of the T_1 values measurement *via* the METALAB software. [7]

Metabolism in in vivo blood: For metabolism analysis, fifteen female healthy BALB/c mice (20 ± 2g, 8-10 weeks) were arbitrarily divided into three groups (n = 5). After injection of **linear and cross-linked PCE-mPEG-Ppa-PROXYL** and 3-CP at a dose of 0.135 mmol PROXYL /kg via the tail vein, 50 μL of blood was collected through the fundus vein at 3 min, 6 min, 9 min, 12 min, 15 min, 30 min, 1 h, 2 h, 4 h, 8 h, 24 h, and 48 h after injection. The collected blood was centrifuged (10000 g × 5 min) to obtain the serum. The nitroxides concentration in the blood was measured by EPR.

In vitro cytotoxicity assay: 4T1 and HUVEC cells were inoculated in 96-well plates at a density of 1×10^4 cells/well. Both **linear and cross-linked PCE-mPEG-Ppa-PROXYL** at different concentrations (5 mg/mL, 2.5 mg/mL, 1.25 mg/mL, 0.625 mg/mL, 0.312 mg/mL, 0.156 mg/mL, 0.078 mg/mL, 0 mg/mL) were added to the DMEM medium to incubate with cells for 24 h. After cleaning twice with PBS, CCK-8 reagent (Dojindo, Japan) of serum-free medium containing was added. After incubating for 2 h in a cell culture box, absorbance was detected by a multifunctional enzyme labeling apparatus. The cell survival rate was calculated

according to the manufacturer's instructions. The cytotoxic effects of **linear and cross-linked PCE-mPEG-Ppa-PROXYL** were compared and evaluated.

Hemolytic Tests: **Linear and cross-linked PCE-mPEG-Ppa-PROXYL** were added to 15 μL of healthy volunteer red blood cell (RBCs) suspensions to obtain different concentrations of polymer solutions (1, 2, and 5 mg/mL). Human blood suspensions incubated with distilled water and PBS (100% lysis) were used as positive and negative controls, respectively. 200 μL of the supernatant was aspirated from all samples to read its absorbance at 540 nm in a 96-well plate by Varioscan Flash.

Effect on RBCs' Morphologies and Aggregation: Fresh blood from healthy human body was extracted to mix with phosphate buffered saline (PBS). The solution was centrifuged (1000 g×5 min) and the supernatant was discarded and the procedure repeated twice to collect RBCs. 2 mg/mL, 2 mg/mL, or 5 mg/mL **linear and cross-linked PCE-mPEG-Ppa-PROXYL** in phosphate buffer solution (PBS) were prepared. 100 μL of each solution was mixed with 20 μL of red blood cells, and the same volume of the PBS solution was used as a control group. After incubation at room temperature for 15 min, the mixture was centrifuged and the supernatant was discarded. 0.5ml 4% PBS paraformaldehyde solution was added to fix RBCs for 4 h. The fixed erythrocytes were resuspend and 20 μL of the suspension was pipetted evenly on the bottom of a 6-well plate, and dehydrated with 75%, 85%, 95%, and 100% ethanol aqueous solution after 5 min. Finally, all samples were dried naturally at a constant temperature of 25 ^{o}C. A scanning electron microscope (SEM) (Manufacturer, Country) was used to observe the morphology and aggregation of RBCs.

Confocal Laser Scanning Microscopy (CLSM): CLSM was employed to observe the entry of two mORCAs into 4T1 cells. 4T1 cells were seeded onto glass plates in a

24-well plate (5×10^4 cells/well) and incubated at 37 ^{o}C for 24 h. 4T1 cells were subjected to **linear and cross-linked PCE-mPEG-Ppa-PROXYL** treatment at a concentration of 1 mg/mL for 1 h, 2 h, and 6 h. After that, 4T1 cells were washed with PBS three times, stained with a DAPI staining solution for 20 min and washed with PBS three times again. The cell plates were fixed with glue on slides. Cells were imaged using a two-photon confocal microscope (Manufacturer, Country).

Histological analysis: After in vivo imaging for 24 h, all animals were sacrificed for in vivo acute toxicity studies. The major organs including heart, liver, spleen, lungs and kidneys were harvested and treated for H&E staining and histological analysis.

Statistical analysis: The student's t-test was used to compare the difference between groups. All reported p values were two-sided with statistical significance at $p < 0.05$ or $p < 0.01$.

Results and Discussion of in vitro degradation

Because the carboxylic acid esters are easy to hydrolyze under the catalysis of esterase, in this study, linear and cross-linked poly(carboxylate ester) were used as macromolecular skeleton to construct mORCAs, in order to obtain good biodegradability. The mORCAs were treated with a classic in vitro carboxylate degradation condition, and GPC was used to detect MW changs, thus displaying their degradation behaviors. According to the results of GPC analysis (Figure S25 and S26, Supporting Information), with the prolongation of incubation time, the original main peaks of high MW of **linear and cross-linked PCE-mPEG-Ppa-PROXYL** gradually decreased, while the peak of low MW gradually appeared and increased, which indicated that the two mORCAs possessed good esterase-sensitive biodegradability.

Results and Discussion of Cellular uptake and Toxicity

We used a confocal laser scanning microscopy (CLSM) to investigate the cell uptake behavior of the PROXYL-based polymeric contrast agents. The result revealed that linear and cross-linked PCE-mPEG-Ppa-PROXYL entered into cells and accumulated in the cytoplasm (red), while they did not enter the nuclei (blue). Meanwhile, the cytoplasmic fluorescence intensity at 6 h was much stronger than that at 1 h and 2 h for linear and cross-linked PCE-mPEG-Ppa-PROXYL (Figure S34, Supporting Information). These fluorescent images suggested that linear and cross-linked PCE-mPEG-Ppa-PROXYL were able to be internalized into cells and distributed in the cytoplasm without entering the nucleus, and the cellular uptake process was time-dependent.

For a new MRI contrast agent for in vivo diagnosis, cytotoxicity is one of important biosafety and biocompatibility indexes which must be assessed in advance. Cytotoxicity of the linear and cross-linked PCE-mPEG-Ppa-PROXYL to 4T1 and HUVEC cells was studied by the CCK-8 assay. As shown in Figure S35 (Supporting Information), both mORCAs at a nitroxides concentration up to 5 μg/mL did not inhibit cell growth for 4T1 and HUVEC cells. There was no significant difference in the cytotoxicity between two cell lines at all nitroxides concentrations (from 0 to 5 μg/mL). The viability of 4T1 cells was equal to or slightly lower than that of normal HUVEC cells. The result suggested that linear and cross-linked PCE-mPEG-Ppa-PROXYL had no toxicity but excellent biocompatibility at the cellular level. Contributions to quite low cytotoxicity of linear and cross-linked PCE-mPEG-Ppa-PROXYL included their nearly neutral surface charge, excellent biodegradability because of hydrolysis of carboxylate bonds in vivo, and low/no toxic organic fragments in their structures.

The hemolysis assay is mainly for detection and quantitation of hemoglobin released from ruptured red blood cells, and hemolysis is a very important biosafety and compatibility index for materials introduced into the body. Hemolysis was evaluated after adding linear and cross-linked PCE-mPEG-Ppa-PROXYL to whole blood, and treatments with pure water and phosphate buffered saline (PBS) were used as controls.

No red color in the supernatants of incubated samples indicated that linear (Figure S36a, Supporting Information) and cross-linked PCE-mPEG-Ppa-PROXYL (Figure S36b, Supporting Information) did not cause hemolysis reaction. In addition, the absorbance of the supernatant samples at 540 nm was measured using a microplate reader to further quantify the extent of hemolysis. The extent of hemolysis was less than 5 % after our experimental contrast agents were incubated for 12 h at 1.0, 2.0 and 5.0 mg/mL with blood cells, indicating they were highly hemocompatible according to the ASTM standard (Figure S36c, Supporting Information). No significant differences were also observed between treatments by PBS and our mORCAs, in this context, they had excellent blood compatibility and reliable biosafety. Very low hemolysis of the linear and cross-linked PCE-mPEG-Ppa-PROXYL was due to their MW ranges and nearly neutral charges on the surface of the material. The linear and cross-linked PCE-mPEG-Ppa-PROXYL did not interact or destroy RBC membrane, and thus had high hemocompatibility.

According to previous reports,[8] the morphology of red blood cells (RBCs) is one of important indicators to assess whether the foreign material causes hemolysis. Due to a neutral surface charge of two mORCAs, they may interact with neutral or positively charged substances in the blood, and the resulting complex may affect the morphology of RBCs. At the same time, the hydrophobic structure in two mORCAs

may also affect RBCs. The morphology of RBCs was observed under a SEM to assess the biological safety of two mORCAs. As shown in Figure S37 (Supporting Information), when the linear (Figure S37a, Supporting Information) and cross-linked PCE-mPEG-Ppa-PROXYL (Figure S37b, Supporting Information) at different concentrations were incubated with RBCs, the morphology of RBCs did not change significantly and there was no aggregation of RBCs. Under a high magnification (1000 ×) of a SEM, the membrane surface of RBCs was smooth and intact with a normal biconcave disc-like structure. There was no obvious structural change compared with the PBS control group. Therefore, both linear and cross-linked PCE-mPEG-Ppa-PROXYL had not induced any changes in the morphology and aggregation of RBCs and thus had great biological safety.

To further evaluate the biosafety of linear and cross-linked PCE-mPEG-Ppa-PROXYL, mice injected with them or 3-CP after 1 day were sacrificed to collect major organs (heart, liver, spleen, lung and kidney), and mice with saline as a control group. The H&E images (Figure S38, Supporting Information) exhibited no toxic effects on major organs after injection of linear (Figure S38d, Supporting Information) and cross-linked PCE-mPEG-Ppa-PROXYL (Figure S38c, Supporting Information) or 3-CP (Figure S38b, Supporting Information), such as necrosis, atrophy, inflammatory infiltration, in comparison with the saline control group (Figure S38a, Supporting Information). These results indicated that linear and cross-linked PCE-mPEG-Ppa-PROXYL at the MR imaging dose had no toxic effects on organs and tissues of living animals.

Supporting Scheme and Figures

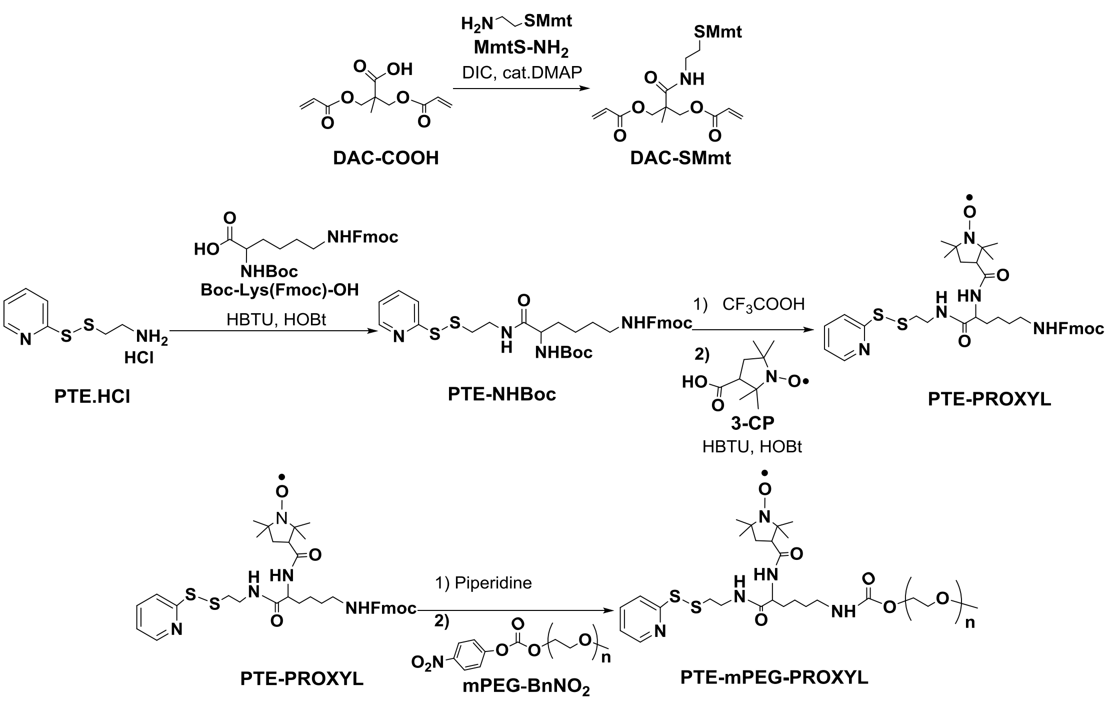

Scheme S1. Synthesis of intermediate products.

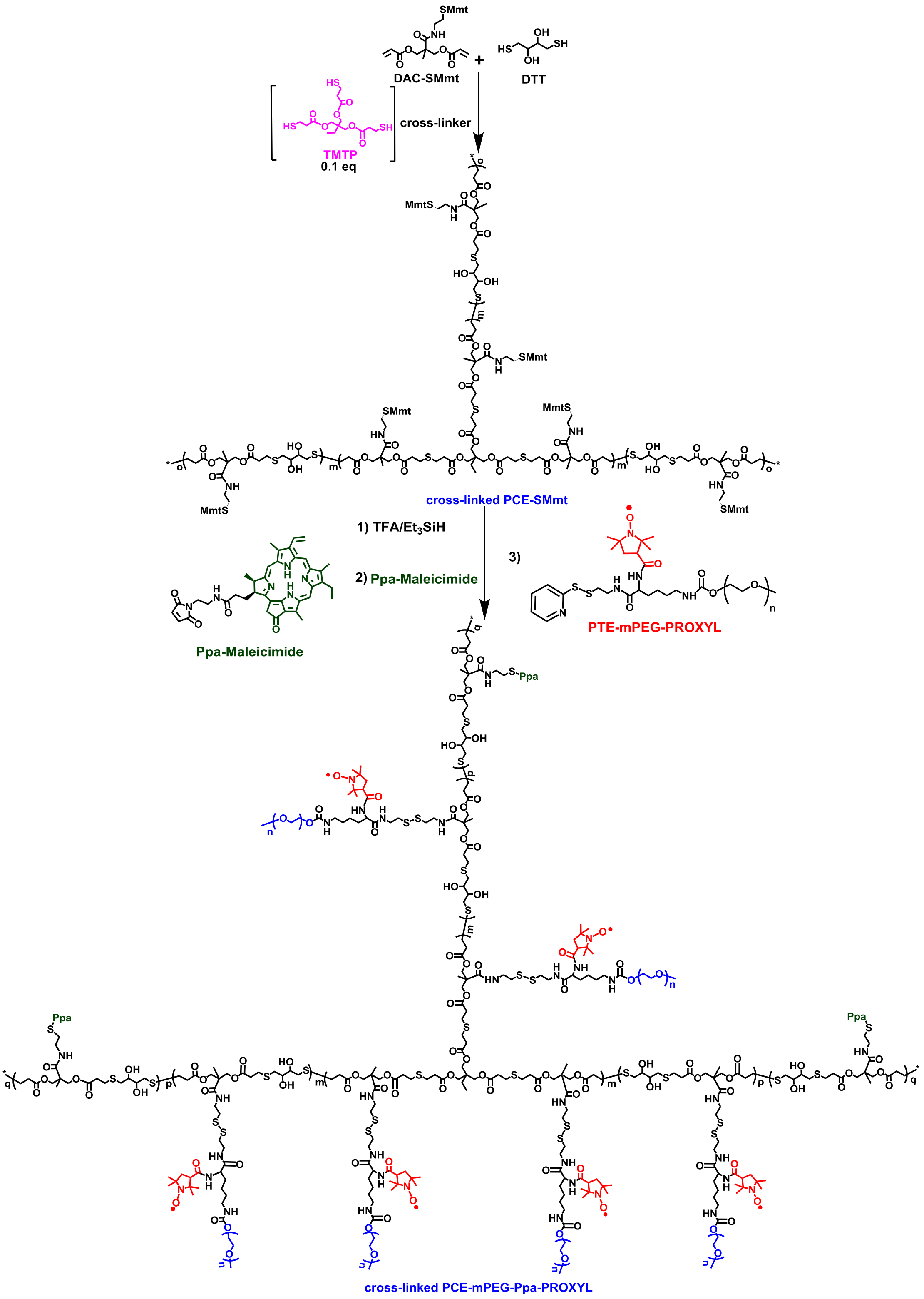

Scheme S2. Synthesis of cross-linked PCE-mPEG-Ppa-PROXYL.

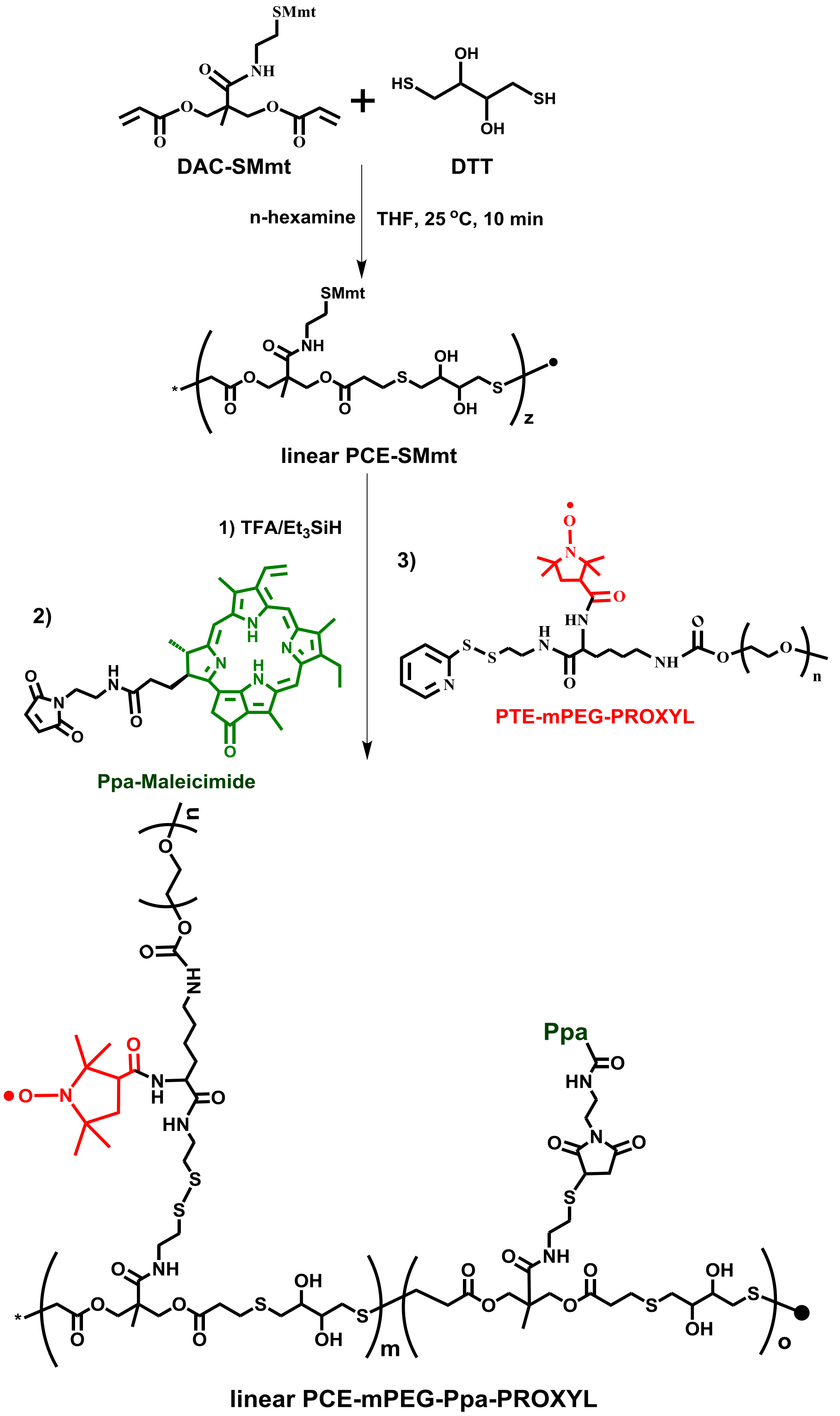

Scheme S3. Synthesis of linear PCE-mPEG-Ppa-PROXYL.

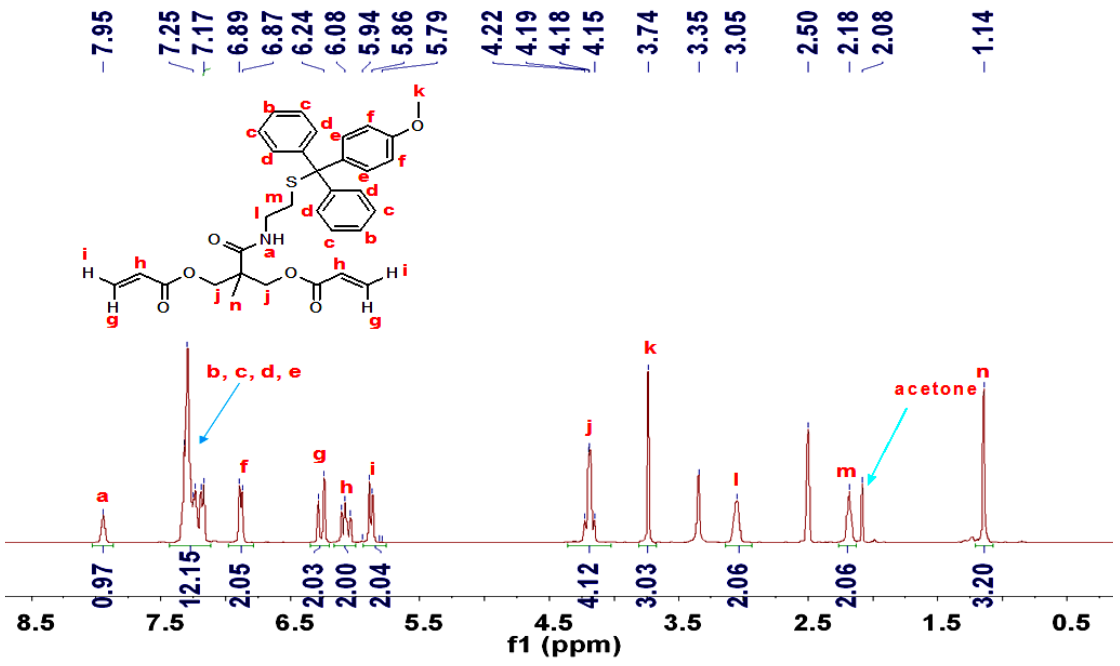

Figure S1. ^{1}H NMR spectrum of DAC-SMmt (recorded in d_6-DMSO).

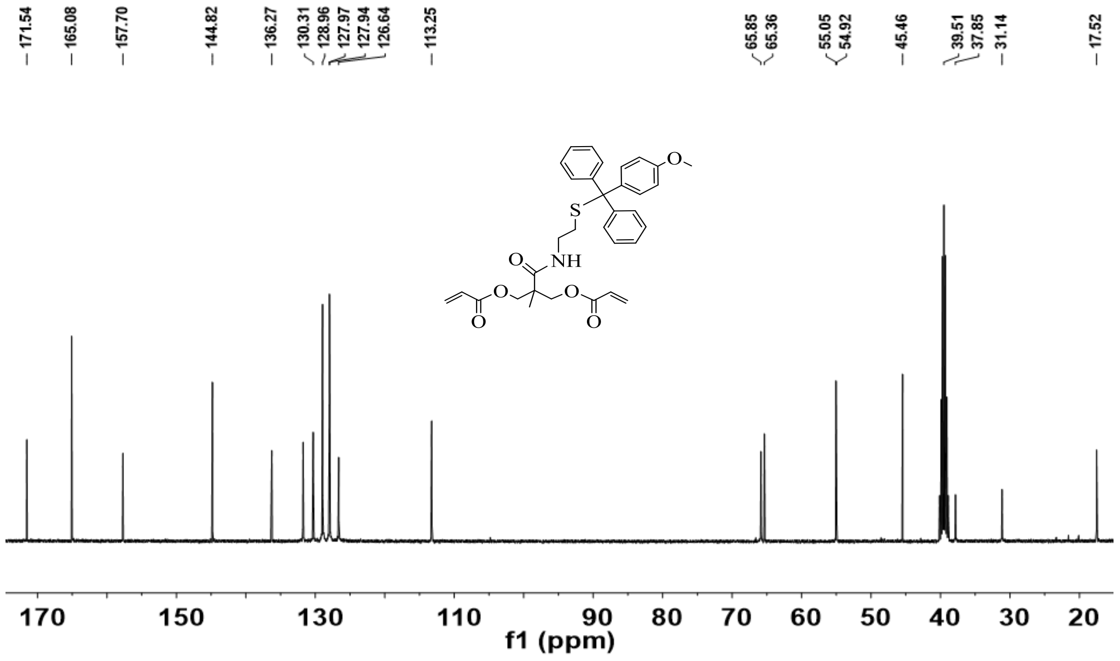

Figure S2. ^{13}C NMR spectrum of DAC-SMmt (recorded in d_6-DMSO).

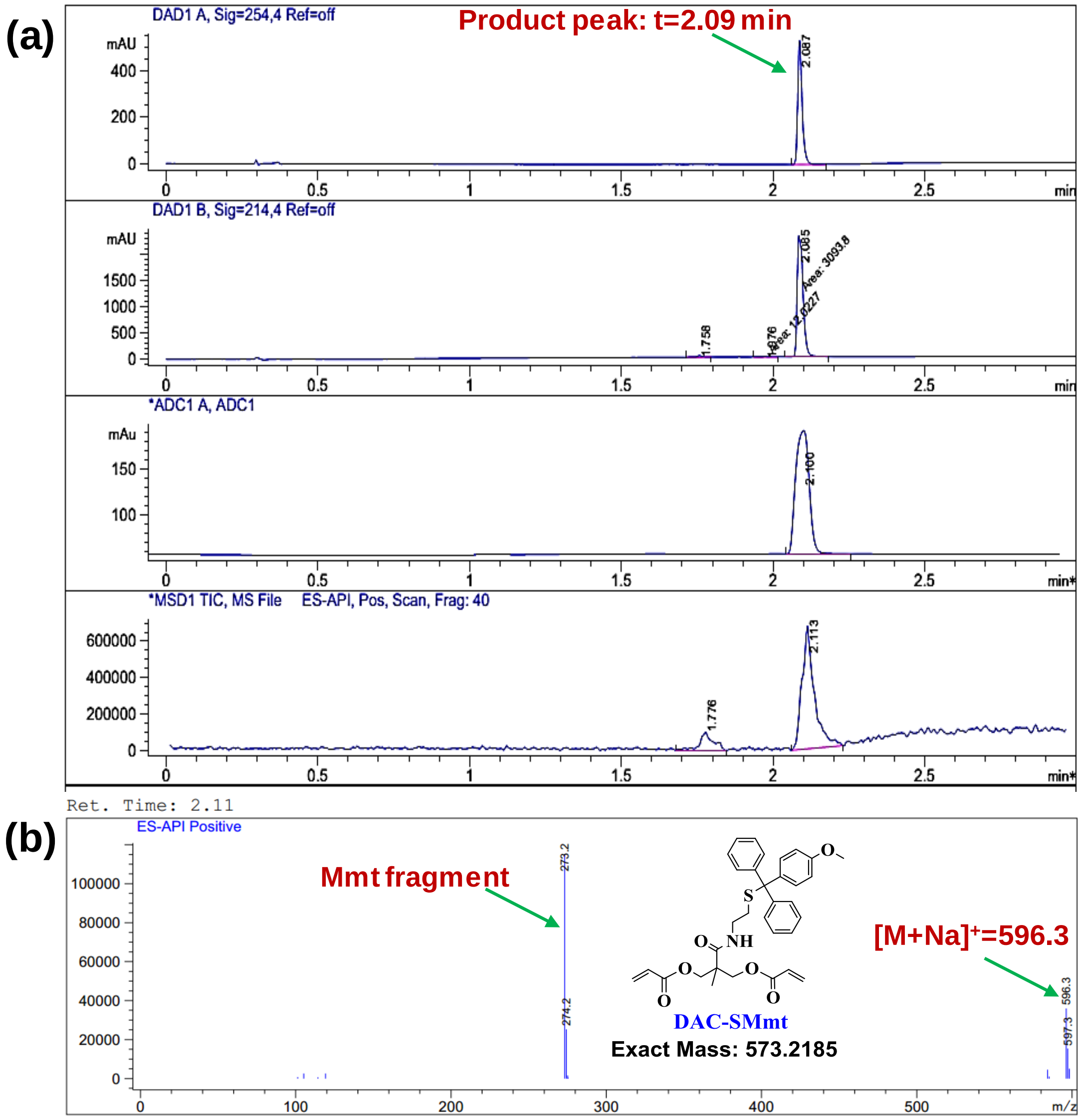

Figure S3. LC-MS spectra of DAC-SMmt. The peak at 2.09 min corresponded to the product peak (a) with a single ion peak at 596.3 m/z for $[M+Na]^{+}$, 273.2 m/z for Mmt fragment (b) (recorded in the acid method).

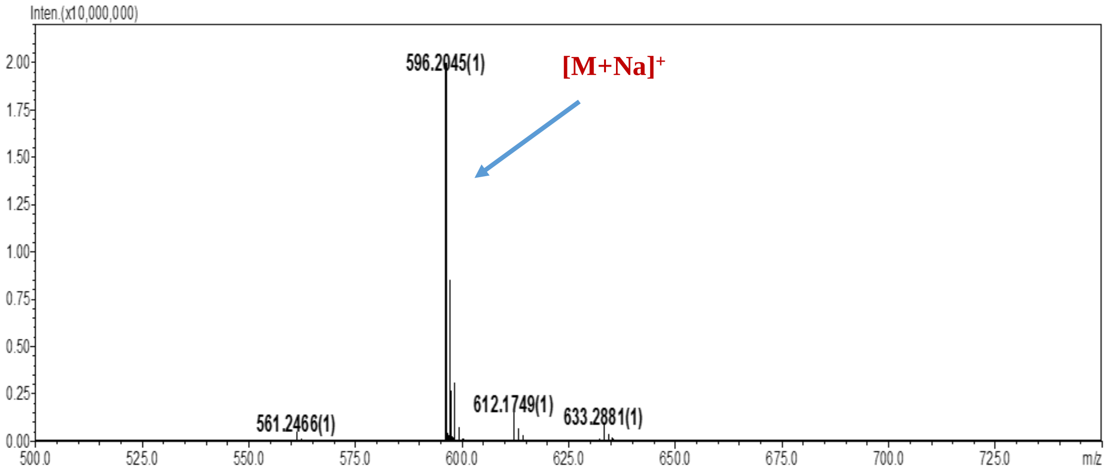

Figure S4. MAIDI-HRMS spectrum of DAC-SMmt, displaying a single ion peak at 596.2045 m/z for [M+Na]$^{+}$.

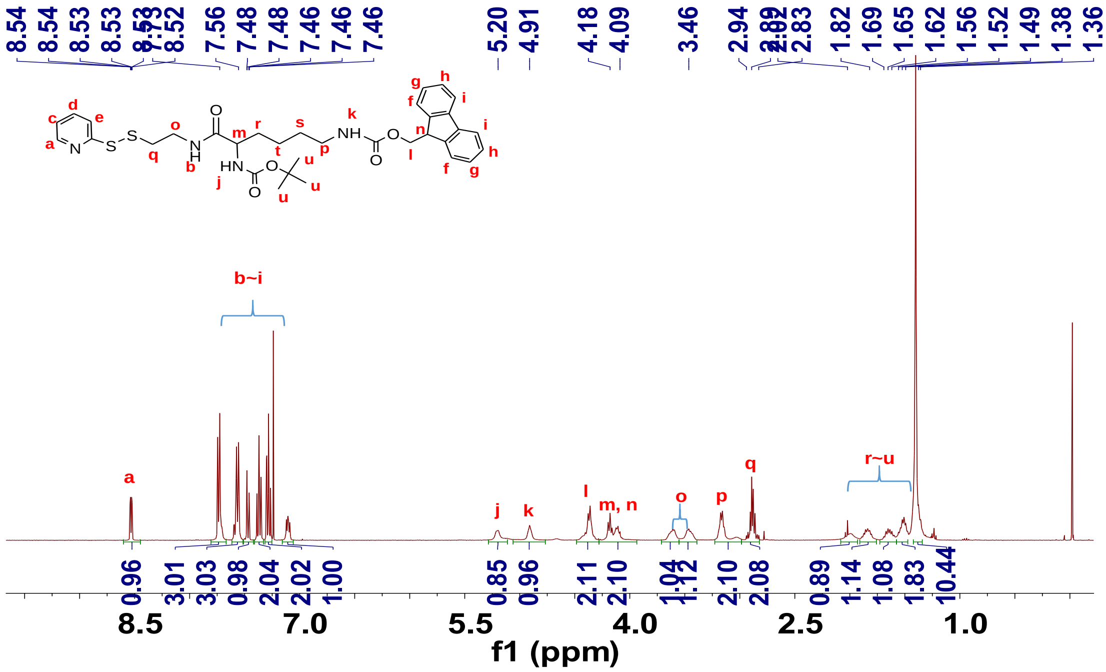

Figure S5. ^{1}H NMR spectrum of PTE-NHBoc (recorded in $CDCl_3$).

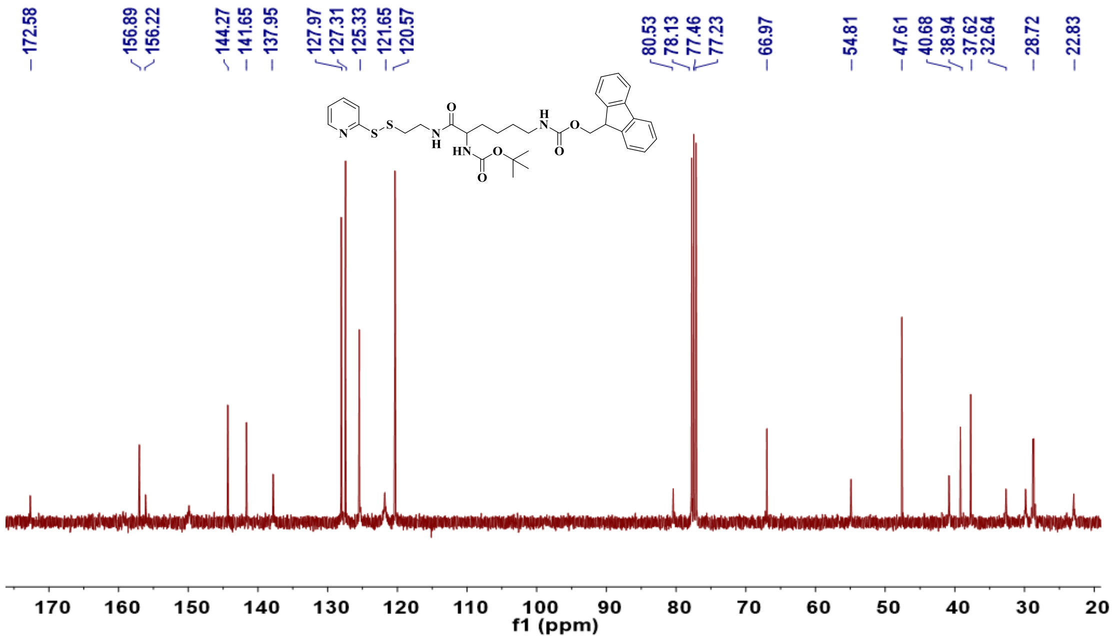

Figure S6. ^{13}C NMR spectrum of PTE-NHBoc (recorded in $CDCl_3$).

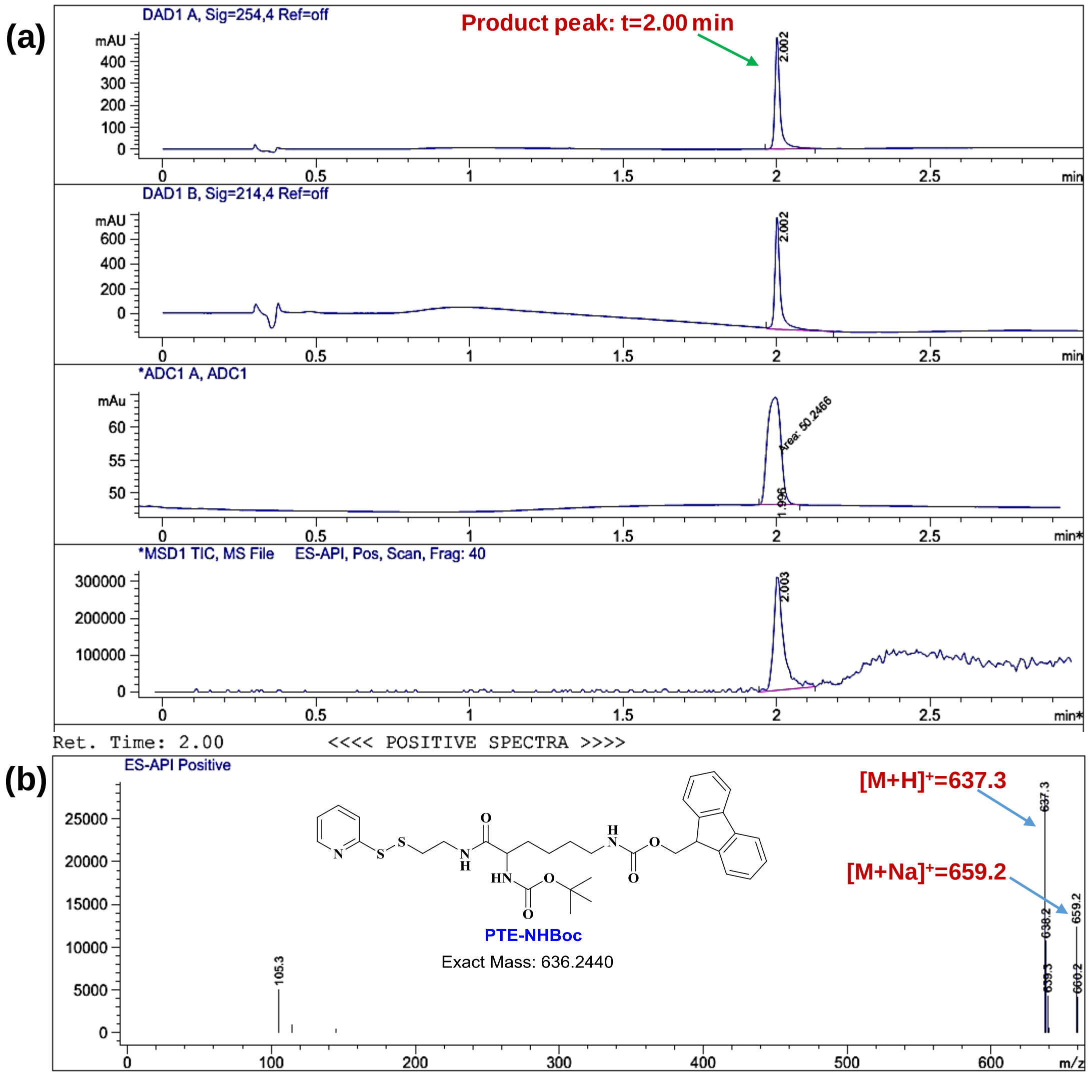

Figure S7. LC-MS spectra of PTE-NHBoc. The peak at 2.00 min corresponded to the product peak (a) with a single ion peak at 537.3 m/z for $[M+H]^+$, 659.2 m/z for $[M+Na]^+$ (b) (recorded in the acid method).

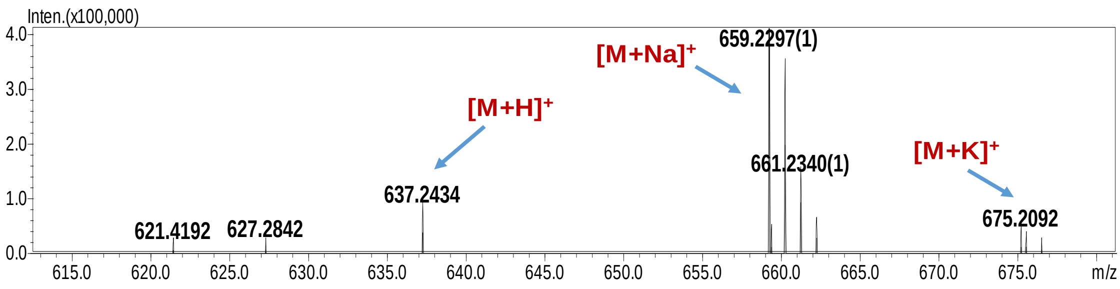

Figure S8. MAIDI-HRMS spectrum of PTE-NHBoc, displaying a single ion peak at 637.2434 m/z for $[M+H]^+$, 659.2297 m/z for $[M+Na]^+$, 675.2092 m/z for $[M+K]^+$.

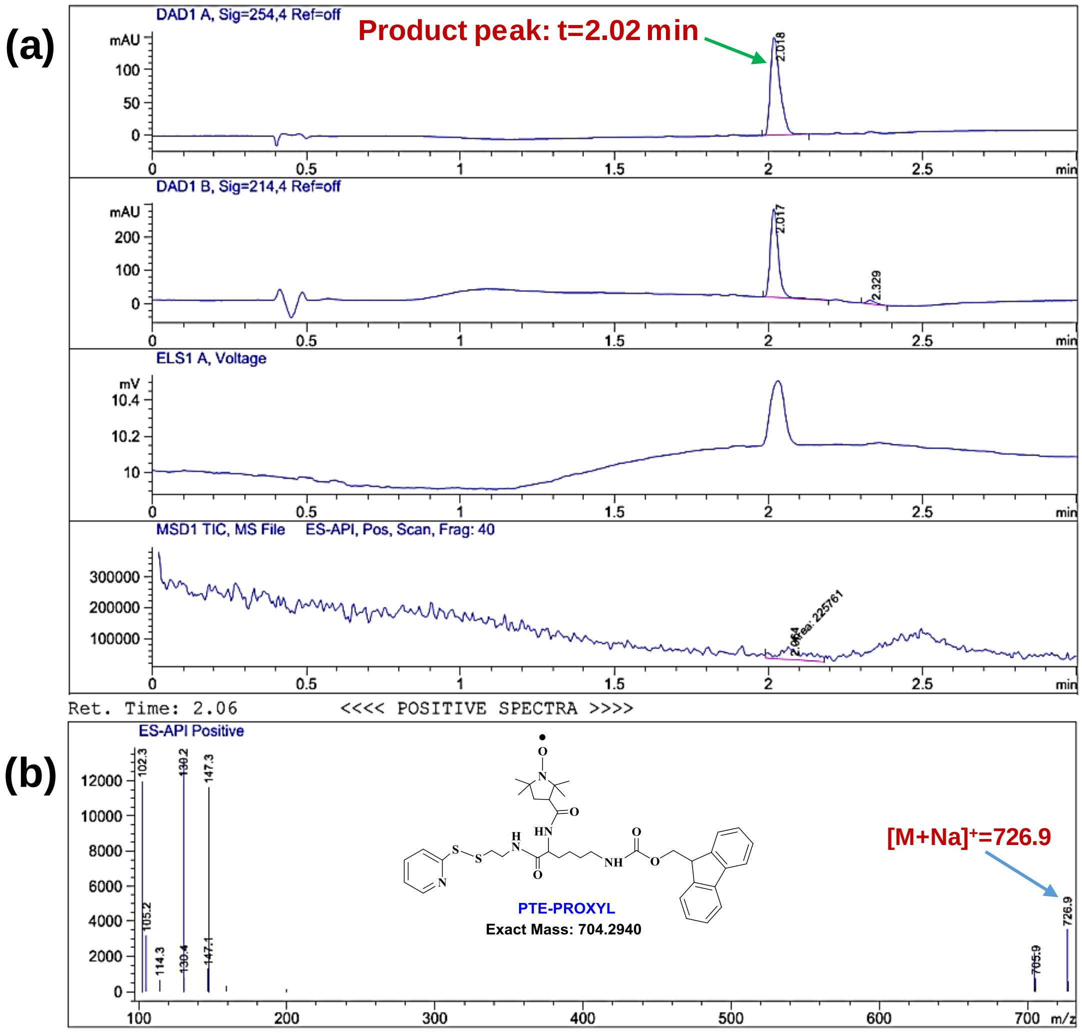

Figure S9. LC-MS spectra of PTE-PROXYL. The peak at 2.02 min corresponded to the product peak (a) with a single ion peak at 726.9 m/z for $[M+Na]^+$ (b) (recorded in the acid method).

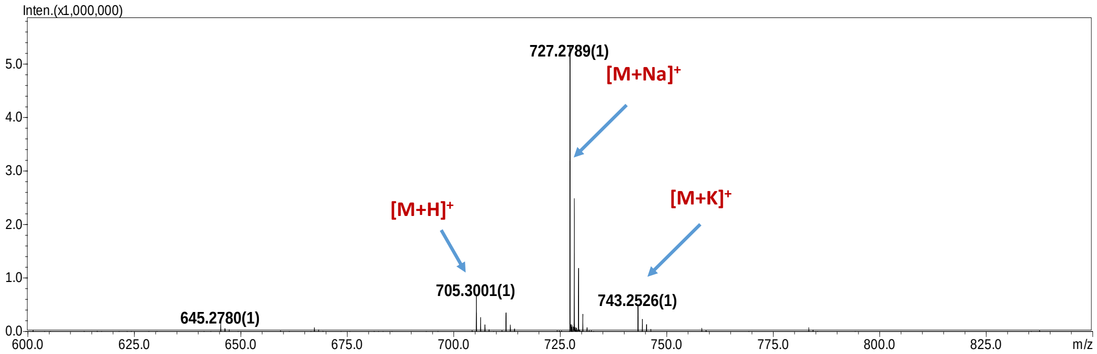

Figure S10. MAIDI-HRMS spectrum of PTE-PROXYL, displaying a single ion peak at 705.3001 m/z for $[M+H]^+$, 727.2789 m/z for $[M+Na]^+$, 743.2526 m/z for $[M+K]^+$.

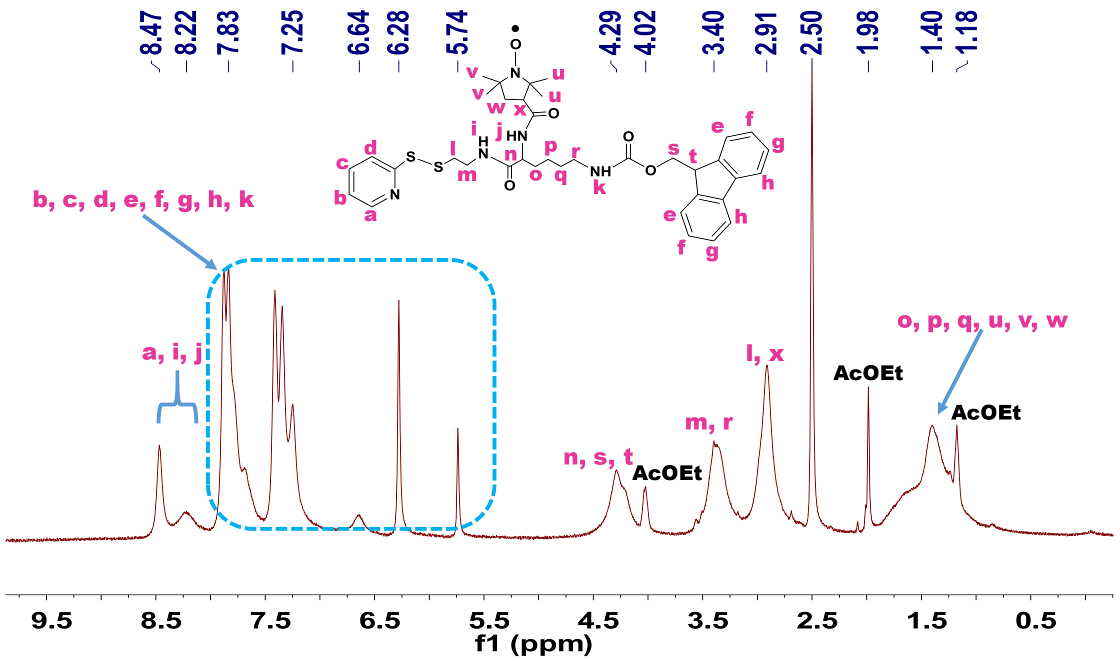

Figure S11. ^{1}H NMR spectrum of PTE-PROXYL(recorded in d_6-DMSO).

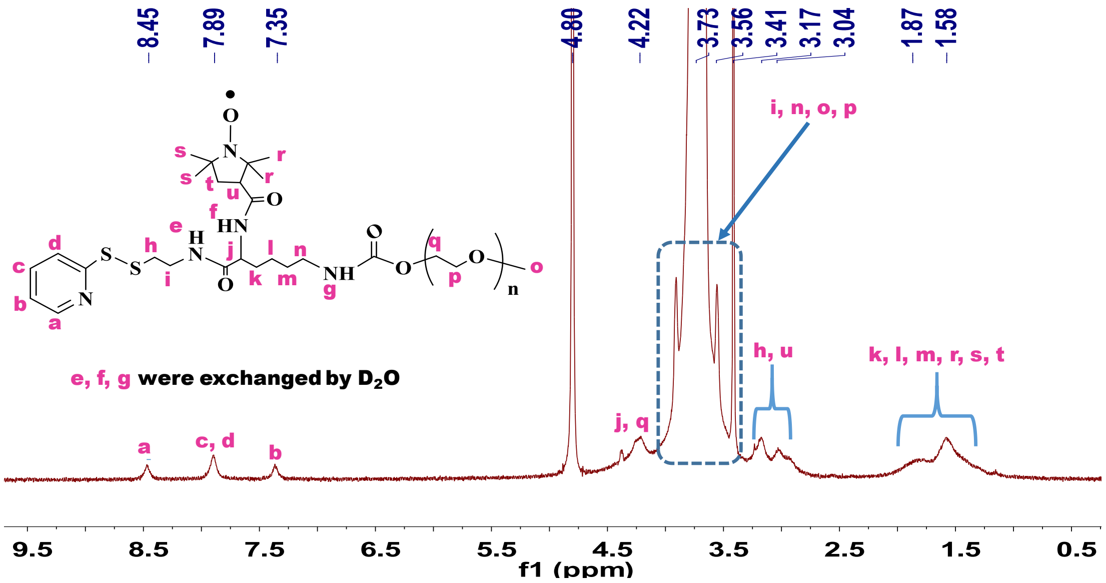

Figure S12. ^{1}H NMR spectrum of PTE-mPEG-PROXYL (recorded in D_2O).

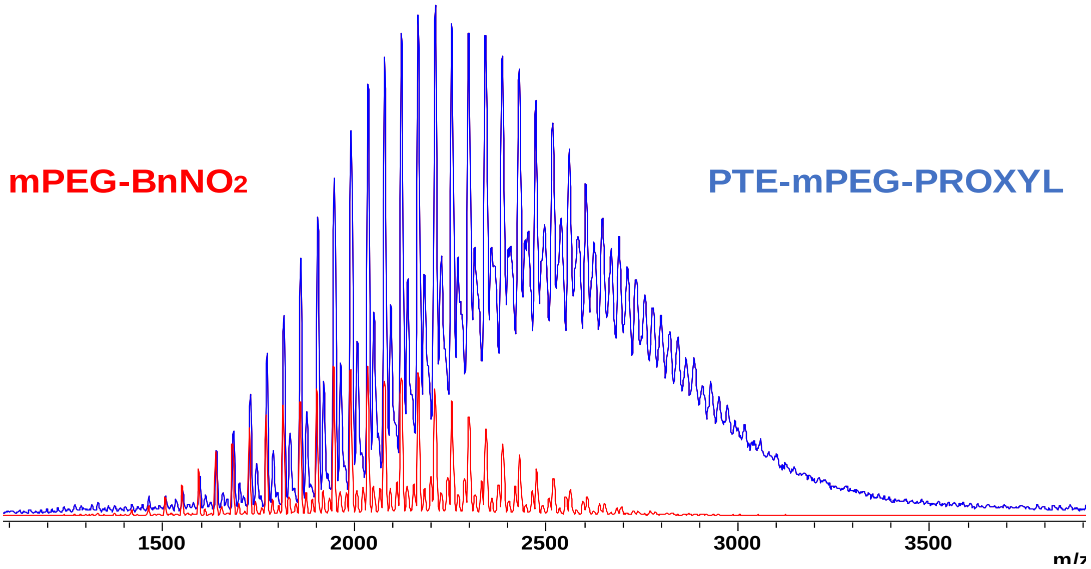

Figure S13. MS spectra of mPEG-NO_2 (red) and PTE-mPEG-PROXYL (blue).

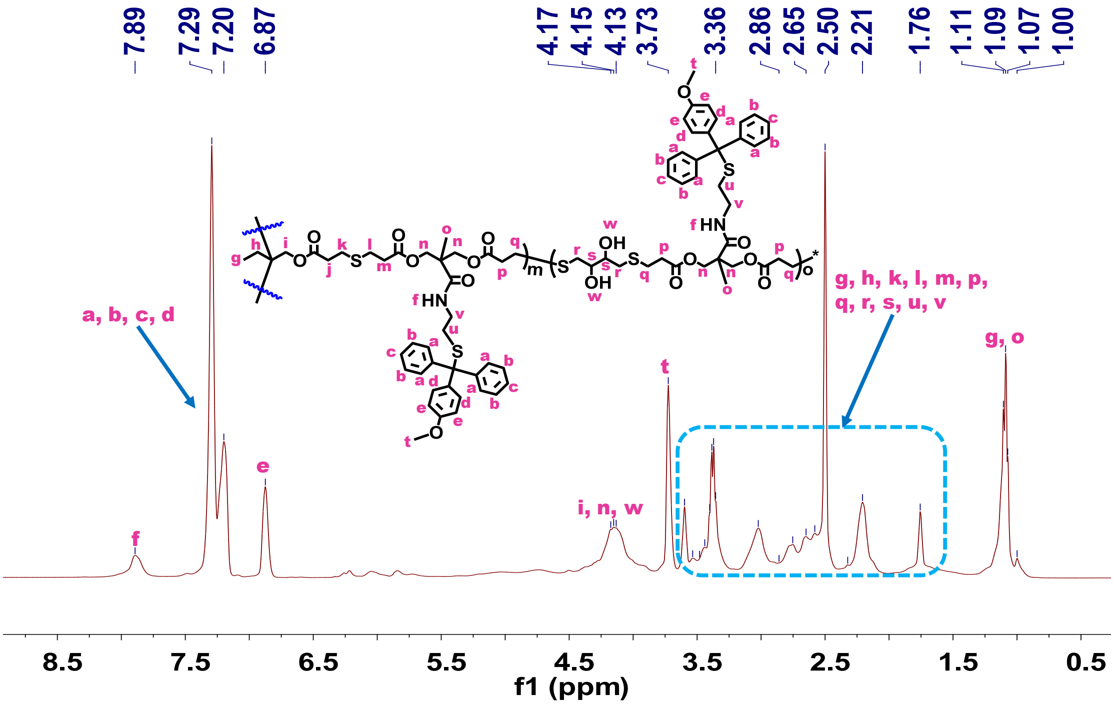

Figure S14. ^{1}H NMR spectrum of cross-linked PCE-SMmt (recorded in d_6-DMSO).

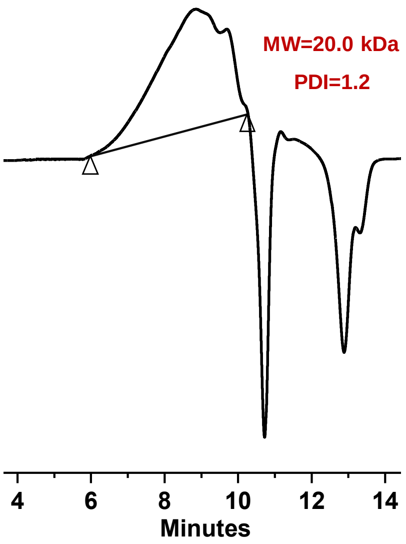

Figure S15. GPC spectrum of cross-linked PCE-SMmt (Waters styragel HT 3 column, DMF as eluent and polystyrene (PS) as standard sample).

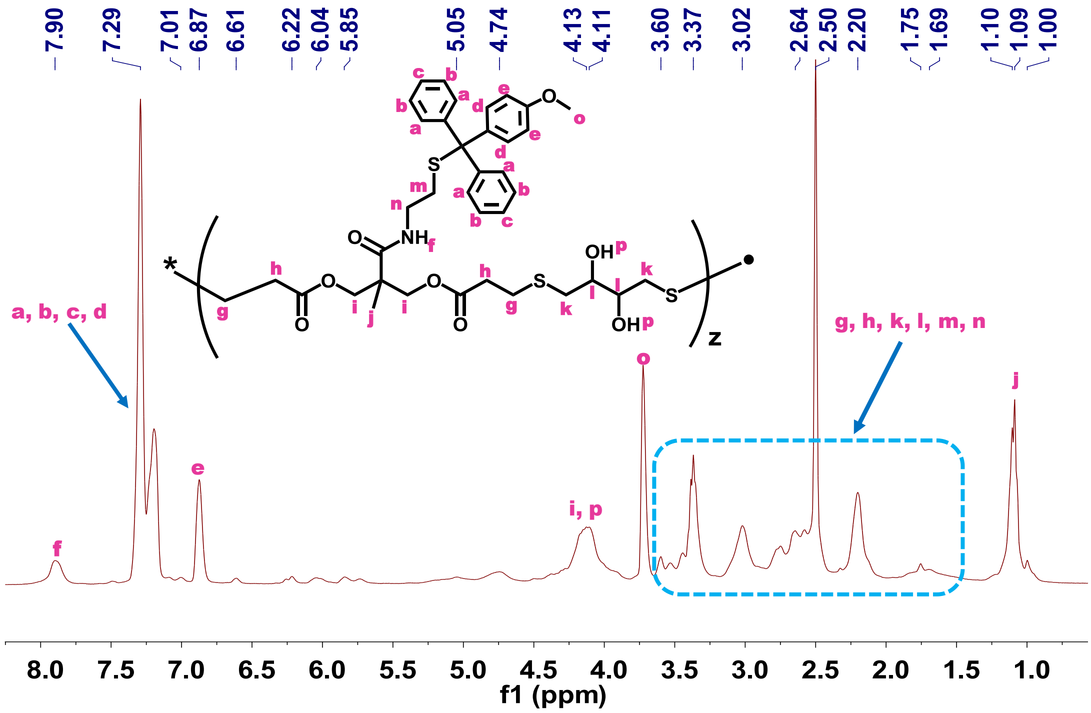

Figure S16. ^{1}H NMR spectrum of linear PCE-SMmt (recorded in d_6-DMSO).

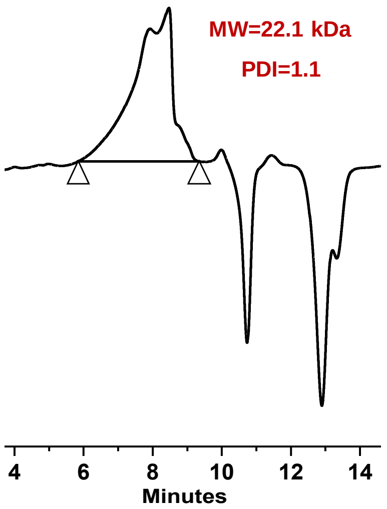

Figure S17. GPC spectrum of linear PCE-SMmt (Waters styragel HT 3 column, DMF as eluent and polystyrene (PS) as standard sample).

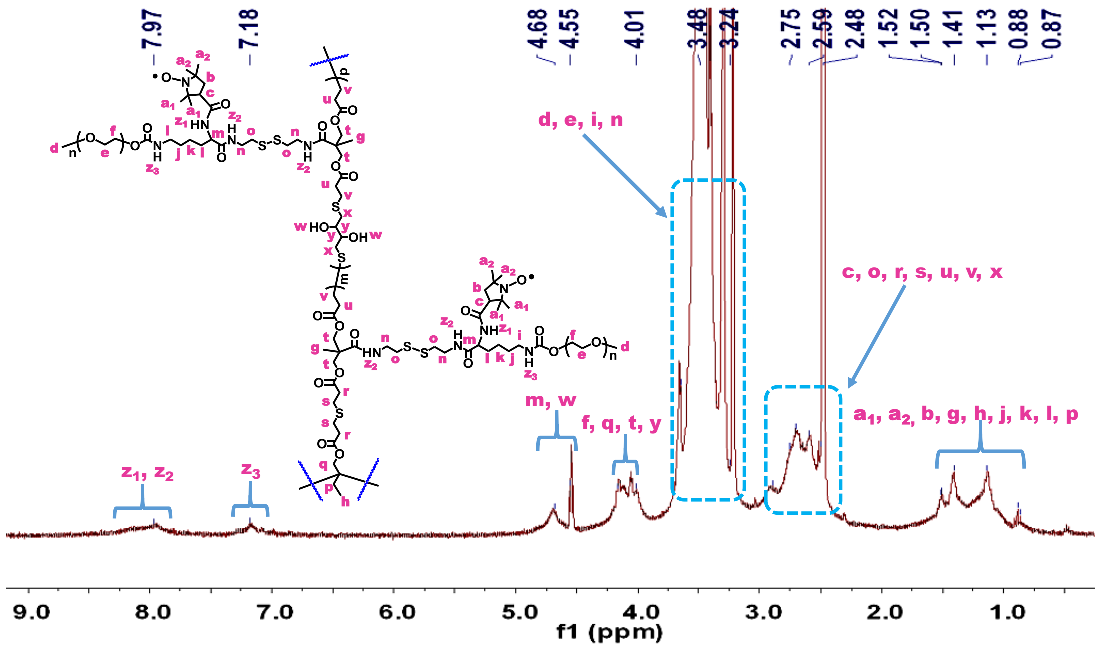

Figure S18. ^{1}H NMR spectrum of cross-linked PCE-mPEG-Ppa-PROXYL (recorded in d_6-DMSO).

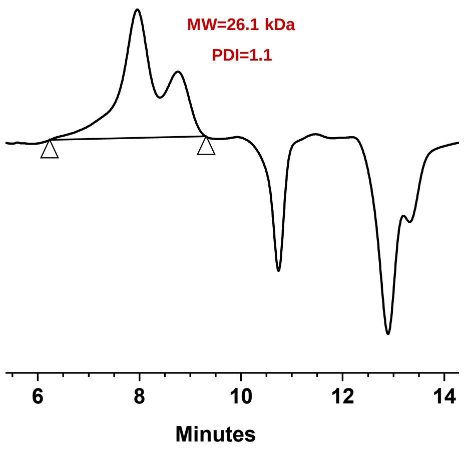

Figure S19. GPC spectrum of cross-linked PCE-mPEG-Ppa-PROXYL.

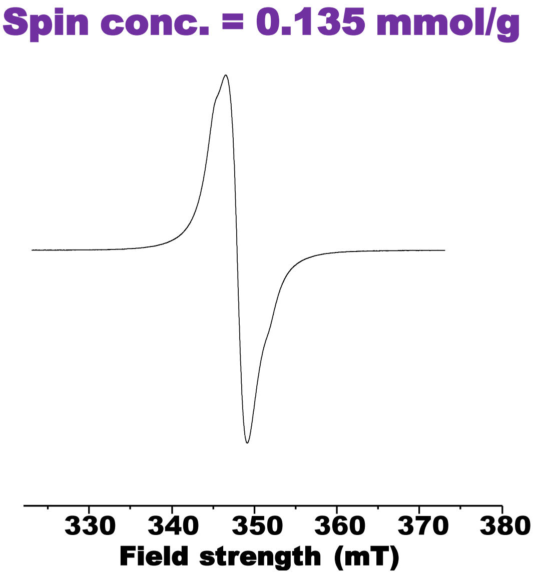

Figure S20. EPR spectrum of cross-linked PCE-mPEG-Ppa-PROXYL.

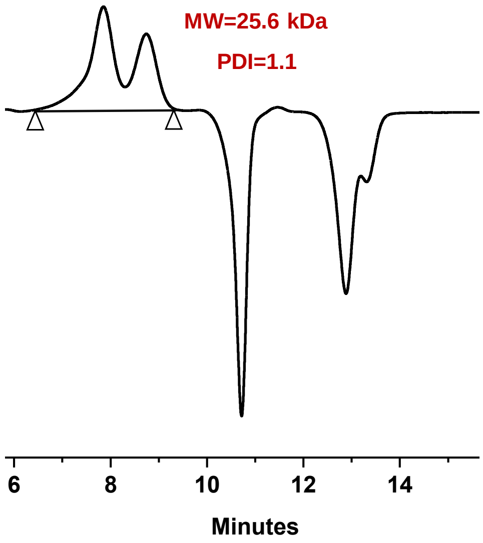

Figure S21. GPC spectrum of linear PCE-mPEG-Ppa-PROXYL.

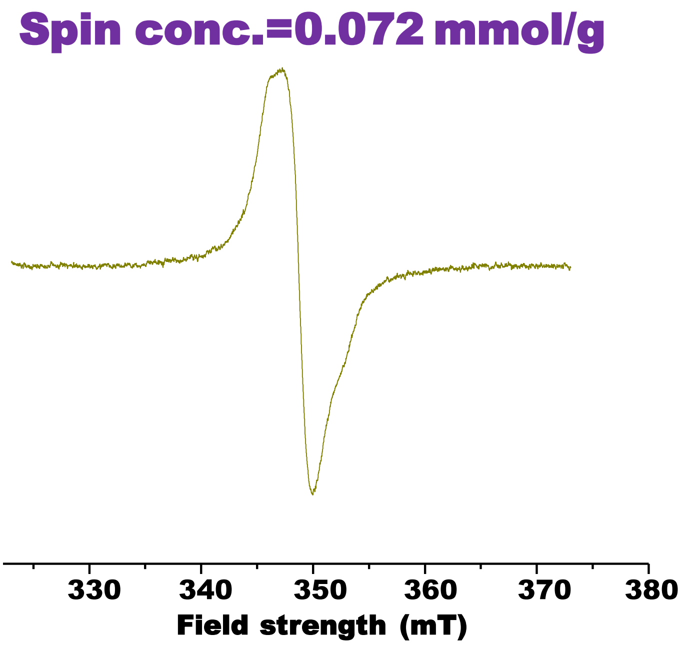

Figure S22. EPR spectrum of linear PCE-mPEG-Ppa-PROXYL.

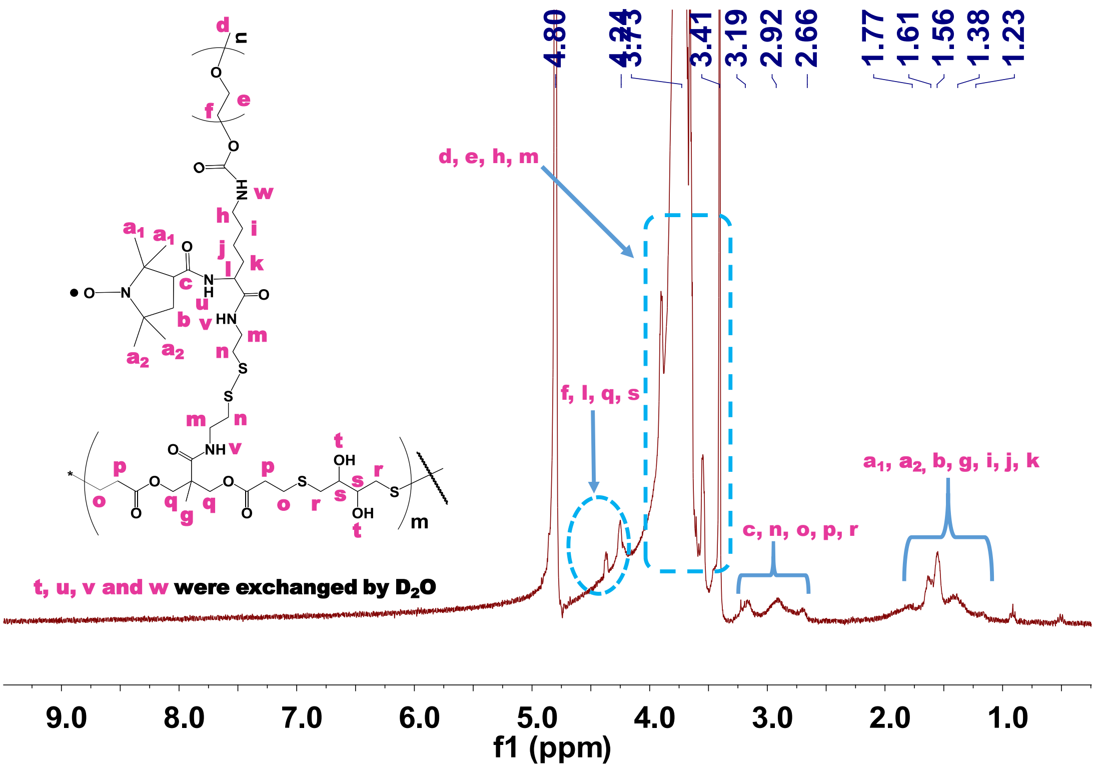

Figure S23. ^{1}H NMR spectrum of linear PCE-mPEG-Ppa-PROXYL (recorded in D_2O).

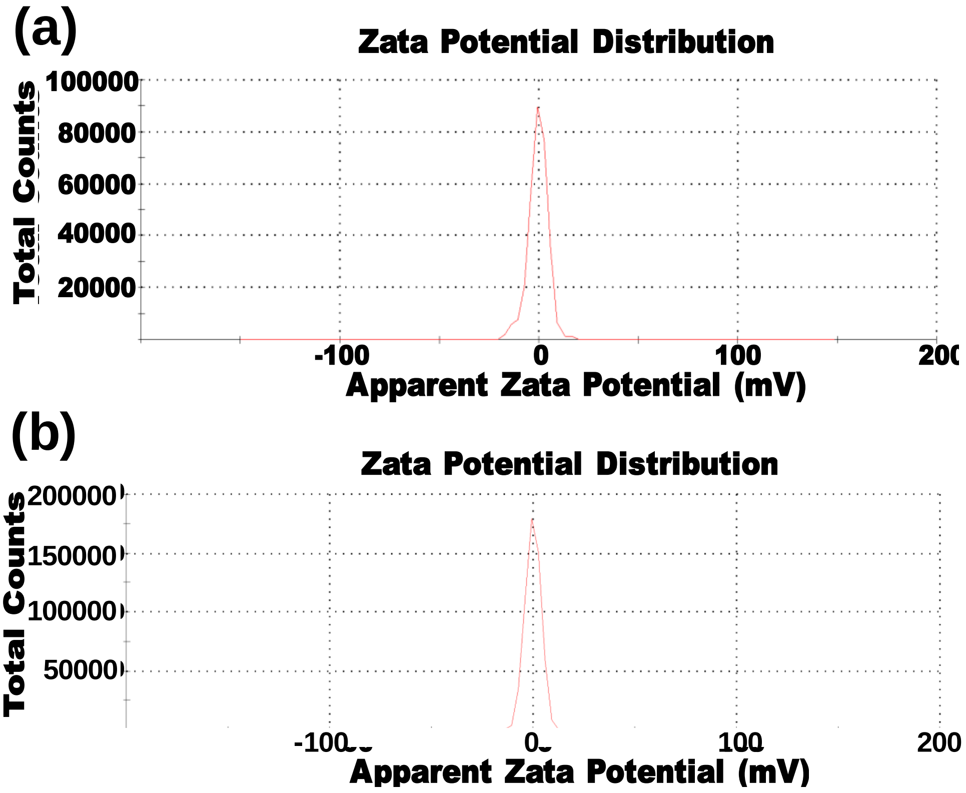

Figure S24. The zeta potential of the linear (a) and cross-linked (b) PCE-mPEG-Ppa-PROXYL (ca. 0 mV).

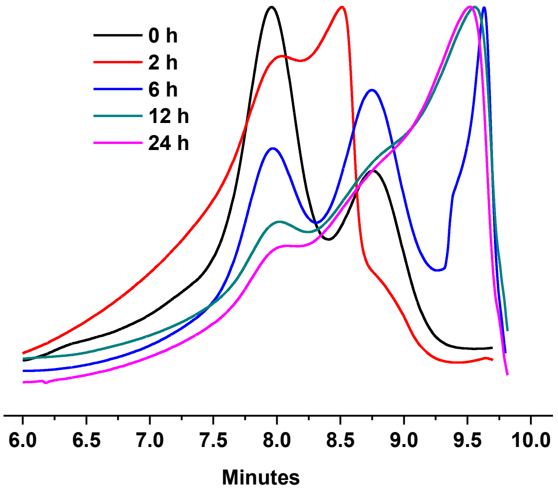

Figure S25. The degradation process of cross-linked PCE-mPEG-Ppa-PROXYL recorded in GPC (Waters styragel HT 3 column, DMF as eluent and PS as standard sample). 1 mg/mL of sample and 200 U of esterase in saline, pH=7.4, 37 °C.

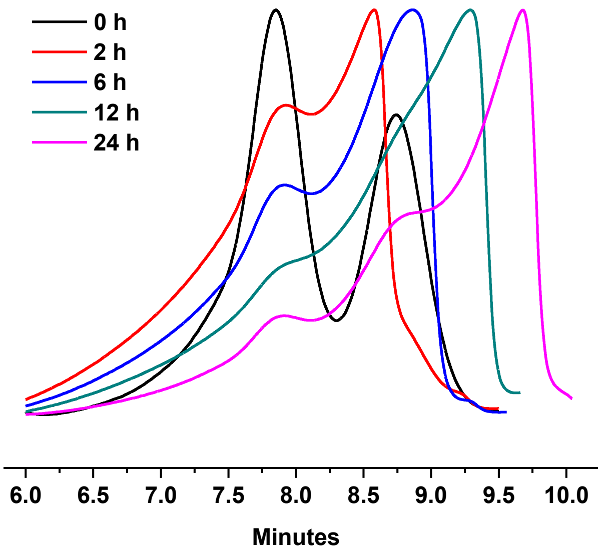

Figure S26. The degradation process of linear PCE-mPEG-Ppa-PROXYL recorded in GPC (Waters styragel HT 3 column, DMF as eluent and PS as standard sample). 1 mg/mL of sample and 200 U of esterase in saline, pH=7.4, 37 ^{o}C.

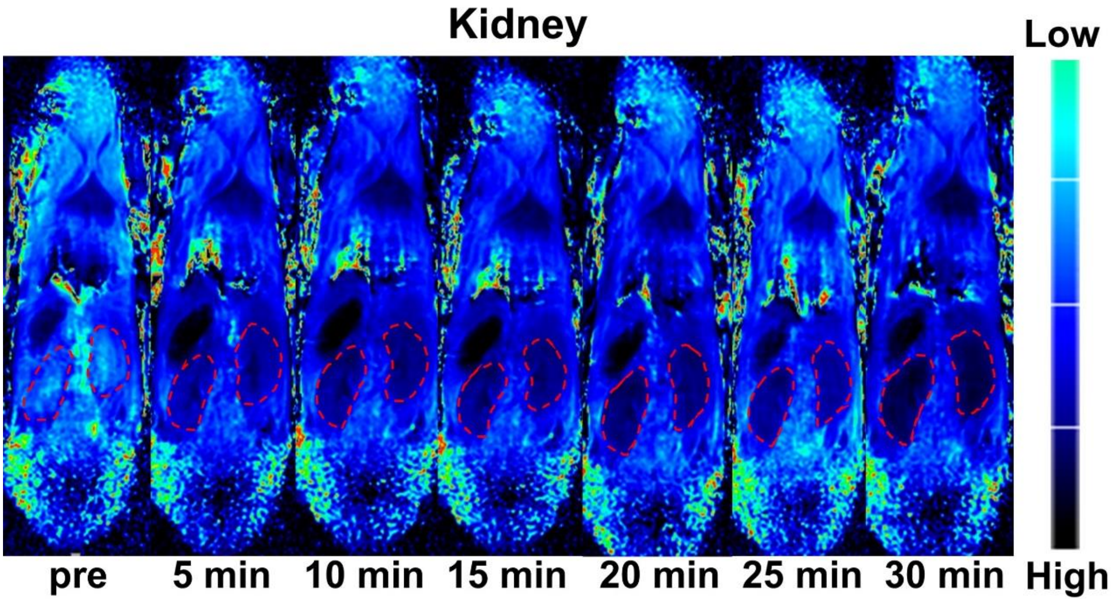

Figure S27. T_1 mapping imaging of kidney after injection of cross-linked PCE-mPEG-Ppa-PROXYL. The kidney was labeled with red dashed lines, and darker blue signals in the kidney suggest sharper enhancement in the MR images.

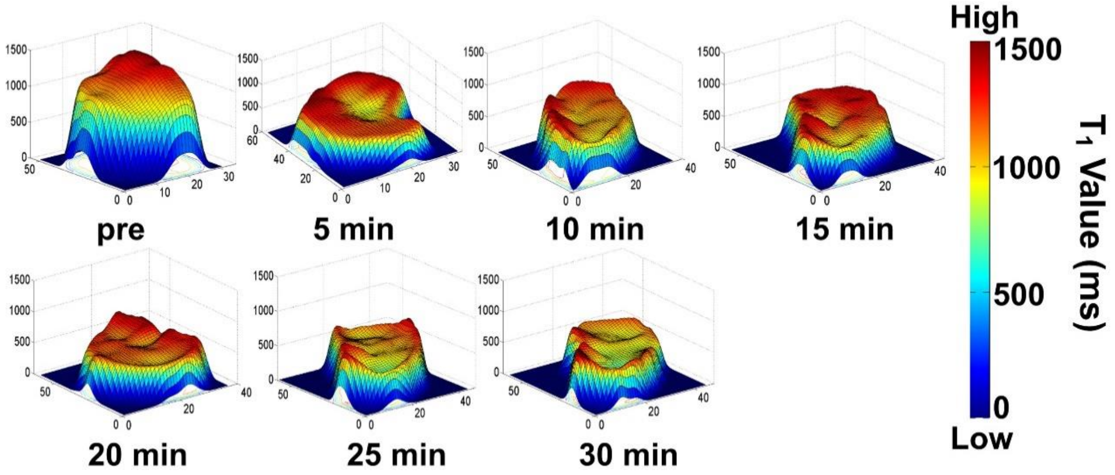

Figure S28. The T_1 values distribution in the kidney after injection of cross-linked PCE-mPEG-Ppa-PROXYL.

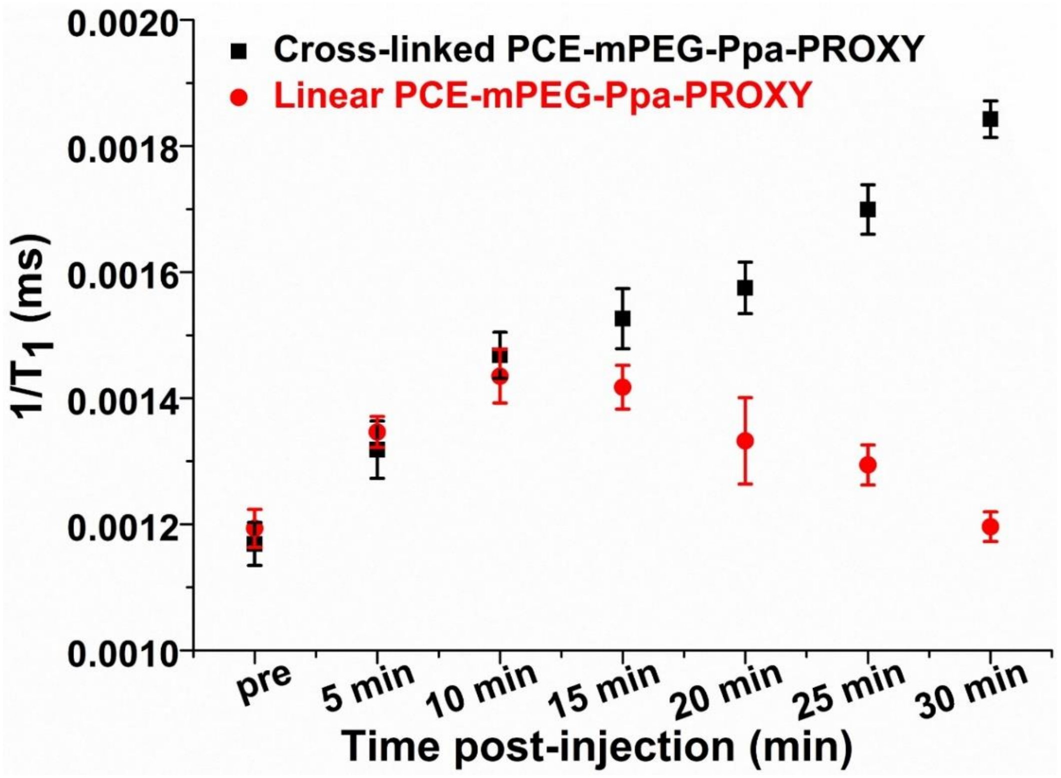

Figure S29. The $1/T_1$ values of two kidney groups were quantitatively analyzed ($p< 0.05$).

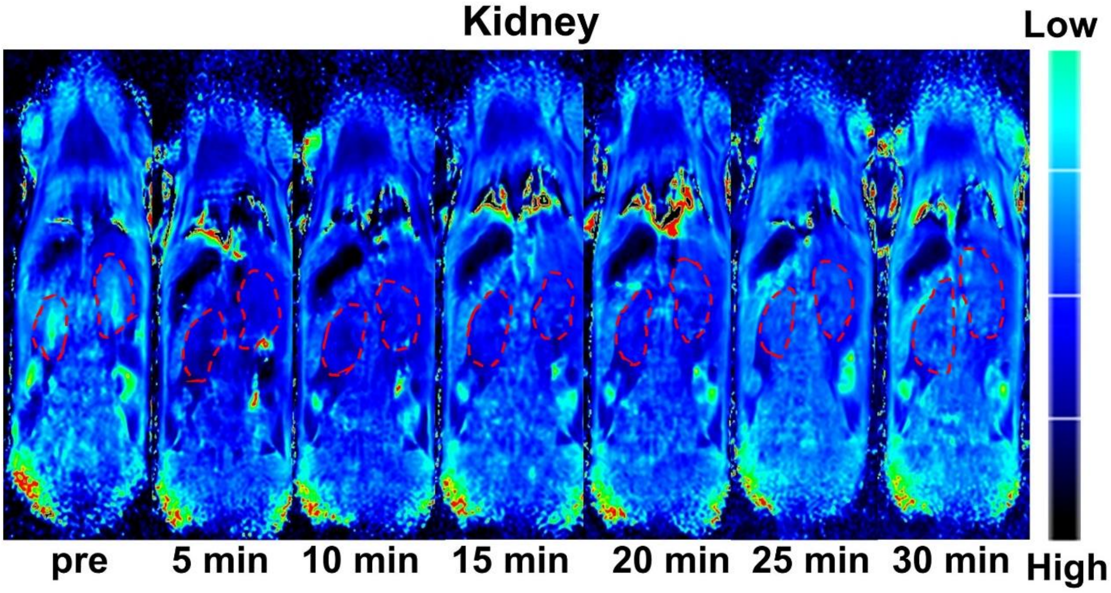

Figure S30. T_1 mapping imaging of kidney after injection of linear PCE-mPEG-Ppa-PROXYL. The kidney was labeled with red dashed lines, and darker blue signals in the kidney suggest sharper enhancement in the MR images.

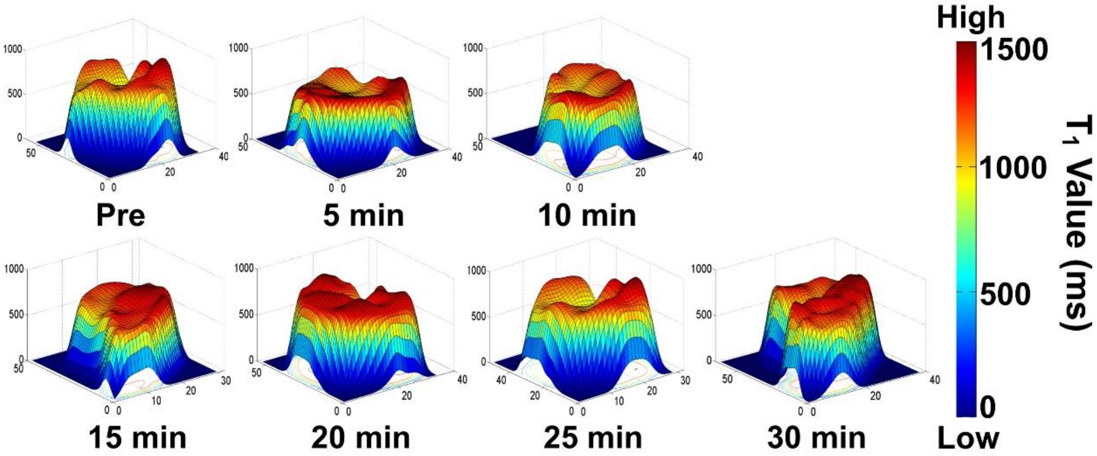

Figure S31. The T_1 values distribution in the kidney after injection of linear PCE-mPEG-Ppa-PROXYL.

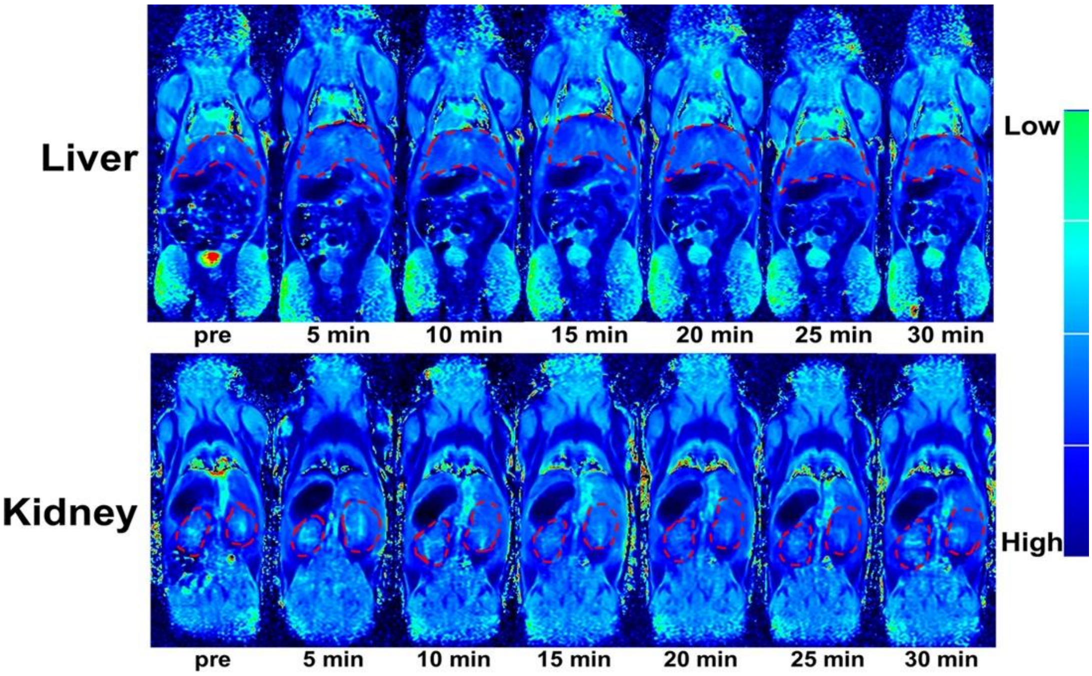

Figure S32. T_1 mapping imaging of liver and kidney after injection of the 3-CP. The liver and kidney were labeled with red dashed line.

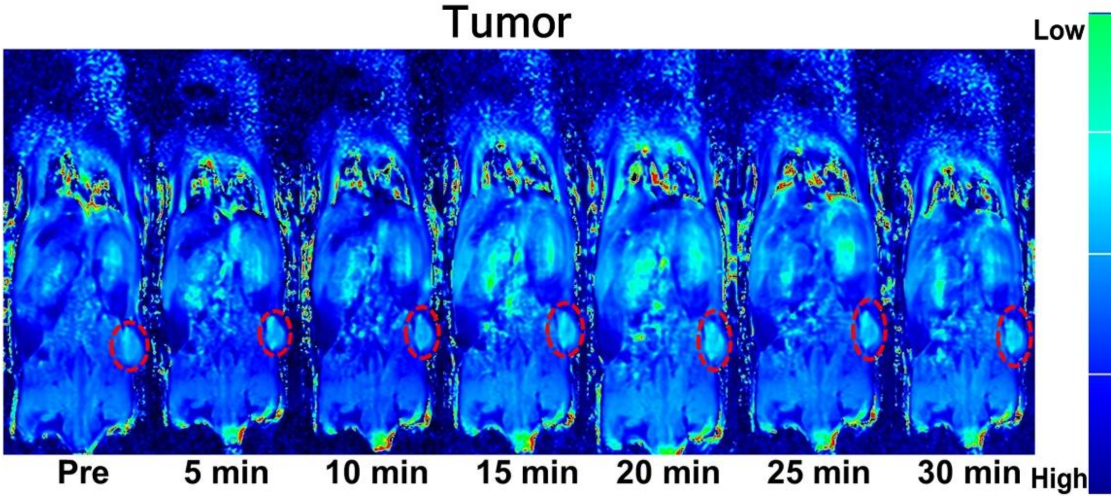

Figure S33. T_1 mapping imaging of tumor by 3-CP. The tumor were labeled with red dashed line circle.

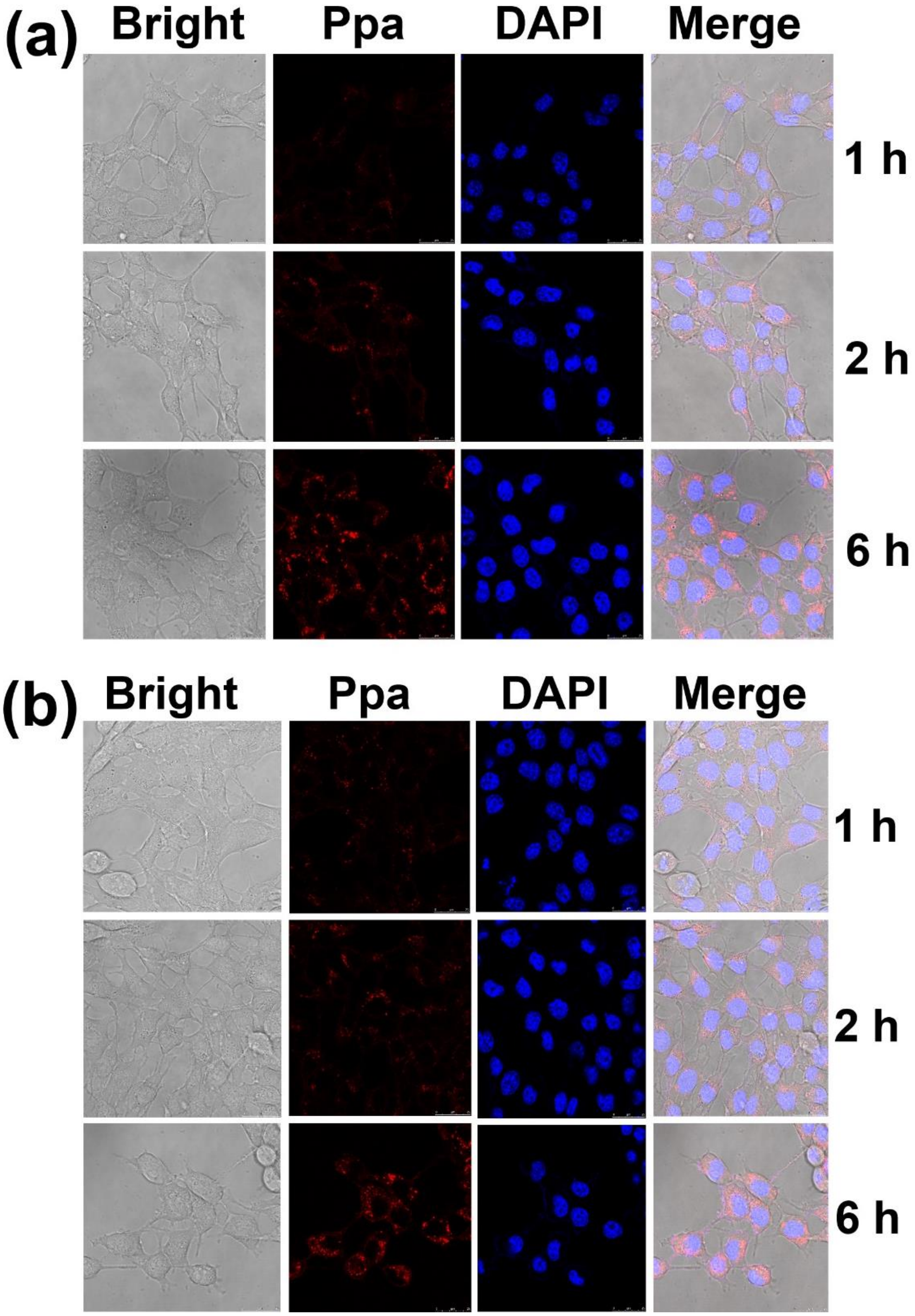

Figure S34. (a) Fluorescence images of 4T1 cells treated with linear (a) and cross-linked PCE-mPEG-Ppa-PROXYL (b) for 1 h, 2 h, 6 h.

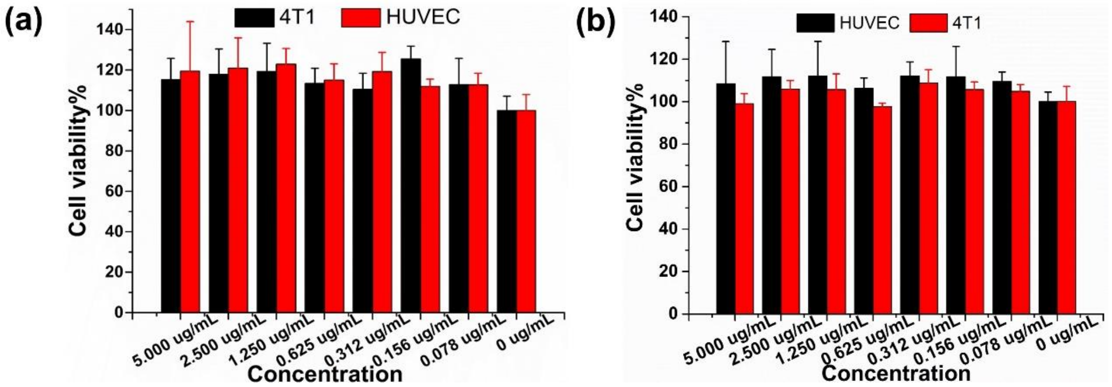

Figure S35. Cytotoxicity of linear (b) and cross-linked PCE-mPEG-Ppa-PROXYL (a) against 4T1 and HUVEC cell lines.

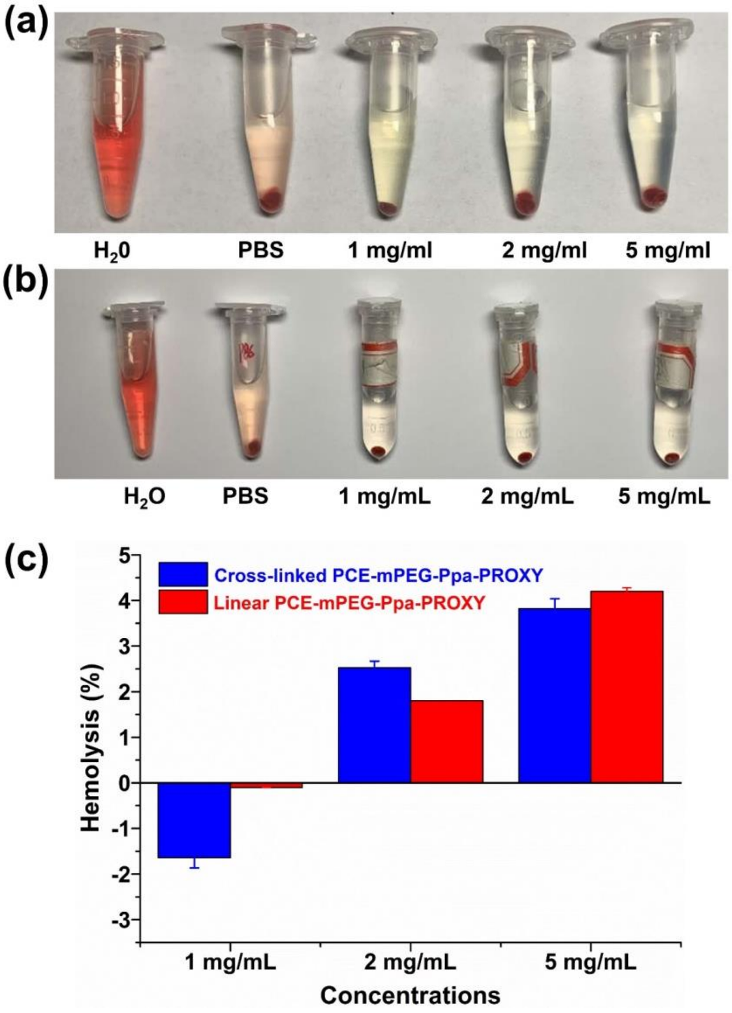

Figure S36. RBC lysis by linear (a) and cross-linked PCE-mPEG-Ppa-PROXYL (b) and (c) quantitative analysis of hemolysis by linear and cross-linked PCE-mPEG-Ppa-PROXYL at various concentrations.

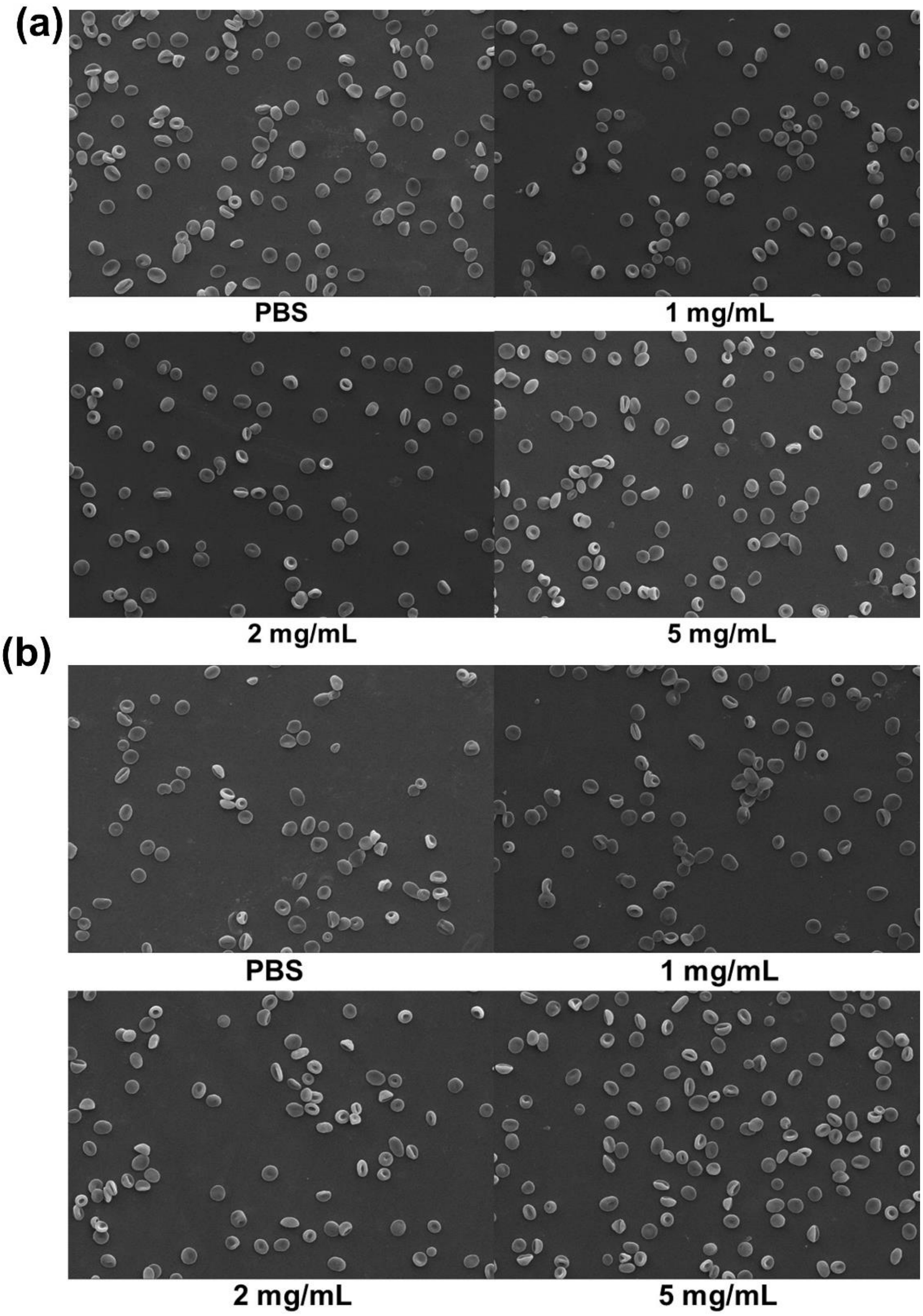

Figure S37. Effect of PBS, linear (a) and cross-linked PCE-mPEG-Ppa-PROXYL (b) at 1 mg/mL, 2 mg/mL, 5 mg/mL on erythrocyte aggregation and morphologies via SEM.

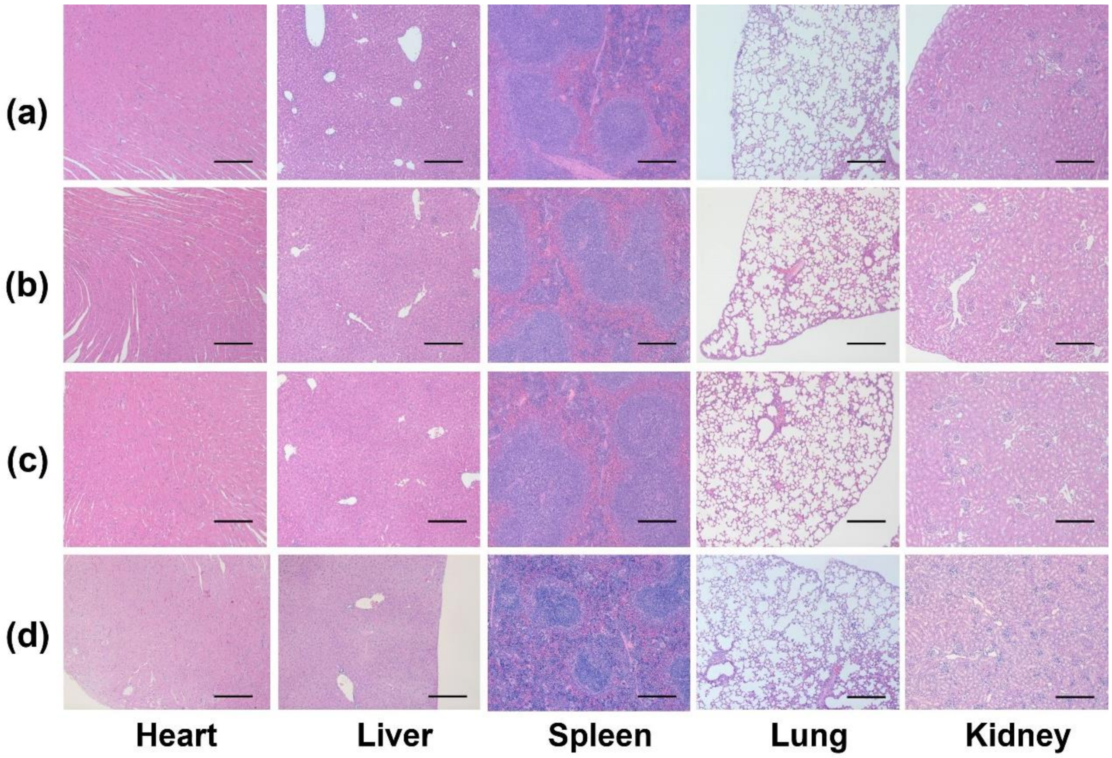

Figure S38. Histological images of major organs of mice administered with saline (a), 3-CP (b), cross-linked PCE-mPEG-Ppa-PROXYL (c) and linear PCE-mPEG-Ppa-PROXYL (d) on day 1 post injection. Scale bar: 10 µm.

References

[1] D. Pan, X. Zheng, Q. Zhang, Z. Li, Z. Duan, W. Zheng, M. Gong, H. Zhang, Q. Gong, Z. Gu, K. Luo, *Adv. Mater.* **2020**, DOI:10.1002/adma.201907490.

[2] X. Ma, Z. Zhou, E. Jin, Q. Sun, B. Zhang, J. Tang, Y. Shen, *Macromolecules* **2013**, *46*, 37.

[3] R.W. Riddoch, P. Schaffer, J.F. Valliant, *Bioconjugate Chem.* **2006**, *17*, 226.

[4] M. Lelle, C. Freidel, S. Kaloyanova, I. Tabujew, A. Schramm, M. Musheev, C. Niehrs, K. Müllen, K. Peneva, *Eur. J. Med. Chem.* **2017**, *130*, 336.

[5] L. Radi, M. Fach, M. Montigny, E. Berger-Nicoletti, W. Tremel, P.R. Wich, *Medchemcomm* **2016**, *7*, 1738.

[6] Z. Zhou, W.J. Murdoch, Y. Shen, *J. Polym. Sci. Pol. Chem*. **2015**, *54*, 507–515.

[7] D. Laurent, J. Wasvary, E. O'Byrne, M. Rudin, *Magn. Reson. Med.* **2003**, *50*, 541.

[8] Q. Luo, X. Xiao, X. Dai, Z. Duan, D. Pan, H. Zhu, X. Li, L. Sun, K. Luo, Q. Gong, *ACS Appl. Mater. Inter.* **2018**, *10*, 1575.
